# Long non-coding RNA ZFAS1 promotes colorectal cancer tumorigenesis and development through DDX21-POLR1B regulatory axis

**DOI:** 10.18632/aging.103875

**Published:** 2020-11-16

**Authors:** Xiufang Wang, Zhikun Wu, Wenyan Qin, Tong Sun, Senxu Lu, Yalun Li, Yuanhe Wang, Xiaoyun Hu, Dongping Xu, Yutong Wu, Qiuchen Chen, Weifan Yao, Mingyan Liu, Minjie Wei, Huizhe Wu

**Affiliations:** 1 Department of Pharmacology, School of Pharmacy, China Medical University, Shenyang 110122, P. R. China; 2 Liaoning Key Laboratory of Molecular Targeted Anti-Tumor Drug Development and Evaluation, Liaoning Cancer Immune Peptide Drug Engineering Technology Research Center, Key Laboratory of Precision Diagnosis and Treatment of Gastrointestinal Tumors, Ministry of Education, China Medical University, Shenyang 110122, P. R. China; 3 Department of Anorectal Surgery, First Hospital of China Medical University, Shenyang 110001, P. R. China; 4 Department of Medical Oncology, Cancer Hospital of China Medical University, Department of Medical Oncology, Liaoning Cancer Hospital and Institute, Shenyang 110042, P. R. China

**Keywords:** RNA helicases, DDX21, lncRNA ZFAS1, POLR1B, colorectal cancer

## Abstract

Increasing evidence supports long non-coding RNA-ZFAS1 as master protein regulators involved in a variety of human cancers. However, the molecular mechanism is not fully understood in colorectal cancer (CRC) and remains to be elucidated. Here, we uncovered a previously unreported mechanism linking RNA helicase DDX21 regulated by lncRNA ZFAS1 in control of POLR1B expression in CRC initiation and progression. Specifically, ZFAS1 exerted its oncogenic functions and was significantly up-regulated accompanied by elevated DDX21, POLR1B expression in CRC cells and tissues, which further closely associated with poor clinical outcomes. Notably, ZFAS1 knockdown dramatically suppressed CRC cell proliferation, invasion, migration, and increased cell apoptosis, which were contrary to the effect caused by ZFAS1 up-regulation. We further revealed that the inhibitory effect caused by ZFAS1 knockdown could be reversed by DDX21 overexpression *in vitro* and *in vivo*. Mechanistically, our research found that ZFAS1 could directly recruit DDX21 protein by harboring the specific motif (AAGA or CAGA). Finally, POLR1B was identified as the downstream target of DDX21 regulated by ZFAS1, which was also up-regulated in CRC cells and tissues and closely related to poor prognosis. The unrecognized ZFAS1/DDX21/POLR1B signaling regulation axis may provide new biomarkers and targets for CRC treatment and prognostic evaluation.

## INTRODUCTION

RNA helicases exert their multiple functions as important modulators and drivers of mRNA metabolism and gene expression relying on ribonucleoprotein complexes forming, transcription factors recruitment, acting as decoys to sequester RNA binding proteins, or directly interacting with proteins by specific binding sites, etc [1–4]. In humans, DEAD-box such as DDX21 (DDX; 21members), the largest ATP-dependent RNA helicase families, are responsible for the regulation of RNA life cycle or protein biogenesis controlling by the RNA polymerase (Pol) I- and/or II-dependent transcriptional manner [5–7]. However, the molecular underpinnings of their involvement, particularly in mammalian cells, remain poorly understood.

Recent accumulating evidence has demonstrated that DEAD-box RNA helicases shared a conserved core structure characterized by nucleic sequence motifs containing Asp-Glu-Ala-Asp [3, 8]. DDX21, the DEAD-box 21 members, harboring the typical motif has been implicated in RNA splicing, ribosome assembly, and translation initiation, which participated in the development and progression of a variety of diseases [7, 9]. A recent study provided evidence that enhancer-mediated enrichment of novel DDX21 and JMJD3 interaction was necessary for nascent transcript synthesis *via* the resolution of aberrant R-loops formation in response to inflammatory stimulus [10]. Notably, recent research confirmed that DDX21 specifically bound to nucleolar small RNA (snoRNA) to localize on nucleoli and then affected the RNA polymerase I (Pol I) complex including POLR1A, POLR1B to regulate rDNA transcription and promoted ribosomal organisms, ribosome biogenesis, and protein translation, thereby promoting cell proliferation in breast cancer [11, 12]. The activity of Pol I is a pivotal determinant for the level of ribosome production and controls cell growth and proliferation. Uncontrolled rRNA generated by Pol I is related closely with aberrant cell proliferation [13, 14]. Another study revealed that significant overexpression DDX21 promoted breast cancer proliferation and development by interacting with c-Jun to activate AP-1 activity, suggesting a critical role of DDX21 in tumorigenesis and development [15]. Thus, DDX21 exerts its functions acting as an RNA helicase, which subjects to the specific regulation of the ATPase activity domains and RNA recognition domains through interactions with a variety of protein partners. Although specific RNA binding domains of DDX21 are responsible for recognizing multiple cellular modulators, no research has investigated the long non-coding RNAs (lncRNAs) to be involved in the context of RNA helicase functions, and subsequently affecting the cell fates decision as well as the underlying molecular mechanism, particularly in cancers. Thus, it is crucial to clarify the interaction pattern and expression regulation between lncRNAs and DDX21 involved in the progression and development of tumors.

LncRNAs are characterized as important modulators of nuclear organization and function, which subsequently serve as structural components to combine with certain proteins, thereby forming the RNA-protein complex, and then regulate the activity of the corresponding protein or change the cellular localization to exert their functions [16–20]. For example, Wang et al. illustrated that lncTCF7 bound to SWI/SNF to promote self-renewal of hepatic cancer stem cells by activating the Wnt signaling pathway [21]. Furthermore, accumulating evidence has demonstrated that dysregulated lncRNAs could function as oncogenes or tumor suppressors so as to play crucial regulatory roles in several processes of cell fate decisions including cell proliferation, cell apoptosis, autophagy, etc [22–24]. LncRNA ZFAS1 (ZNFX1 antisense RNA 1), located on chromosome 20q13, abnormally expressed in a variety of cancers including breast cancer, hepatocellular carcinoma (HCC), gastric cancer, non-small cell lung cancer (NSCLC), etc [25, 26]. It was found for the first time that ZFAS1 served as a tumor suppressor in breast cancer [27, 28]. In contrast in other solid tumors, ZFAS1 acted as an oncogene to promote the development and progression of cancers such as HCC, NSCLC, etc [25, 29]. In gastric cancer, ZFAS1 simultaneously interacted with EZH2 and LSD1/CoREST to inhibit KLF2 and NKD2 transcription to exert its oncogenic effects [30]. Furthermore, ZFAS1 increased VEGFA expression by binding to miR-150-5p *via* activating EMT and Akt/mTOR signaling pathways to promote the development of colorectal cancer [31]. Despite the emerging knowledge regarding the roles of lncRNA ZFAS1 in cancers, the expression landscape, regulation network, and function manner with target proteins such as DDX21/POLR1 family have not been investigated in regard to colorectal cancer.

Here, we discovered a previously unreported molecular mechanism regulated by lncRNA ZFAS1-DDX21-POLR1B signaling axis involved in CRC initiation and pathogenesis. Mechanistically, the core member of RNA helicase DDX21 was targeted by ZFAS1 in a positive linear correlation upon direct binding manner, fine-tuning the expression of its downstream critical target, the POLR1B (a key subunit of Pol I complex), and then subsequently promoted cell proliferation, invasion, migration and reduced cell apoptosis *in vitro* and *in vivo*. Thus, our findings shed new light on understanding the molecular regulation manner of ZFAS1 in CRC tumorigenesis and provided promising targets for the CRC diagnosis and treatment.

## RESULTS

### Association of lncRNA-ZFAS1 and DDX21 expression in CRC cells and tissues

**Figure 1:**
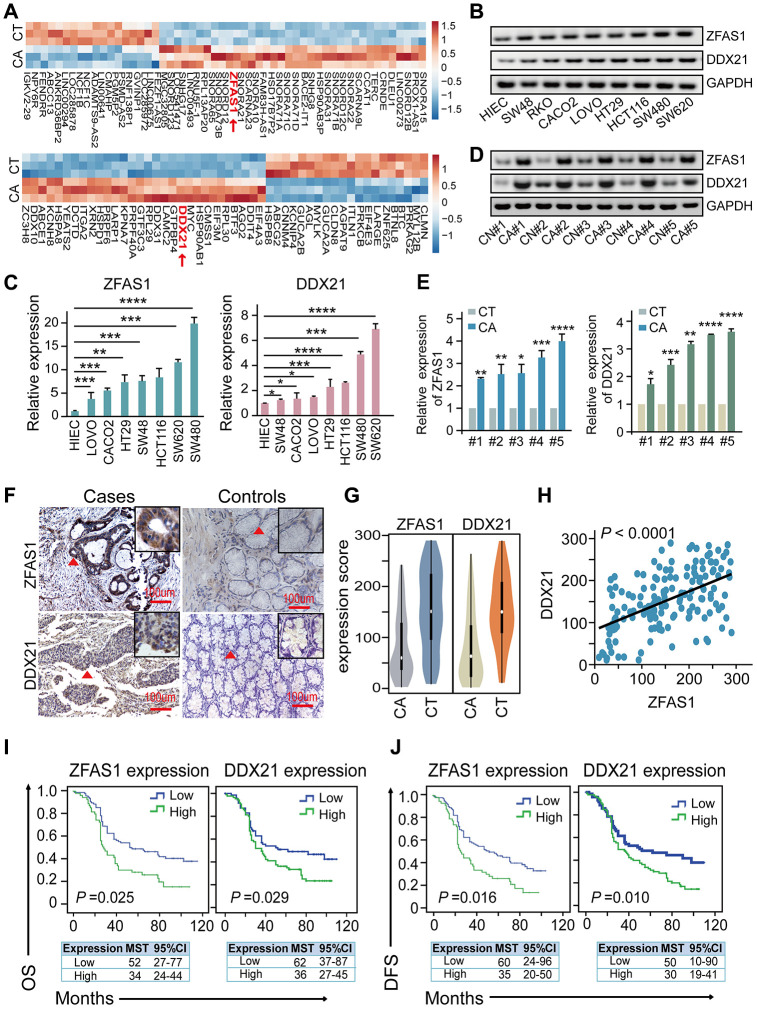
**The association of lncRNA ZFAS1 and DDX21 expression in CRC cells and tissues.** (**A**) The hierarchical clustering heat map illustrating the most differentially expressed lncRNAs and mRNAs in CRC patient tissues and their matched paired adjacent-tumor samples (*n* = 3), selected top 20 up-regulated or down-regulated genes (*P*<0.05). Red in heat map denotes up-regulation. Blue denotes down-regulation. (**B** and **C**) The expression levels of lncRNA ZFAS1 and DDX21 in normal intestinal epithelial HIEC cell and CRC cells including SW480, SW620, SW48, HCT116, RKO, CACO2, LOVO, and HT29 cells detected by RT-PCR (**B**) and qPCR assays (**C**). GAPDH was selected as an internal control. (**D** and **E**) Representative data of lncRNA ZFAS1 and DDX21 expression in paired CRC and matched adjacent-tumor controls detected by RT-PCR and qPCR assays (5 representative data was shown). (**F**) ISH method detected the cellular localization and the expression of lncRNA ZFAS1, and IHC assay determined the DDX21 expression based on this included CRC patient tissues and matched tumor-adjacent controls (*n* = 157). The bar represents 100μm. (**G**) Violin charts displaying the expression levels of lncRNA ZFAS1 and DDX21 in this included CRC cohort. Nonparametric tests and median (interquartile range) were shown. (**H**) Linear correlation pattern showing a positive relationship between the expression of lncRNA ZFAS1 and DDX21. (**I** and **J**) Kaplan-Meier plot curves showing the association of lncRNA ZFAS1 high/low expression, DDX21 high/low expression with the OS (**I**) and DFS (**J**) in this included CRC patients. * *P* <0.05; ** *P* <0.01; *** *P* <0.001; **** *P* <0.0001.

To explore the dysregulated *lncRNAs-mRNAs* enrichment and their correlations, we performed differential expression profiling analysis between CRC patients’ tissues and matched tumor-adjacent control tissues (*n* = 3) upon *Affymetrix GeneChip* microarray dataset that obtained the accession number GSE137511 (https://www.ncbi.nlm.nih.gov/geo/query/acc.cgi?acc=GSE137511). We firstly detected the significant differences between the two groups by fold change ≥ 2 and *P* value < 0.05, as illustrated in Figure 1A and Supplementary Table 8. Specifically, we demonstrated that lncRNA-ZFAS1 was remarkably up-regulated (log_2_ Fold Change/FC = 6.65) in the CRC tissues. Thereafter, we detected the potential target indicators including DDX21, DDX10, DDX31, IGTA2, and LAMC2 in CRC cells by qPCR assay (Supplementary Figure 1A). Importantly, DDX21 was the most significant decreased after ZFAS1 knockdown in both SW620 and SW480 CRC cells, shown in the Supplementary Figure 1A, and the log_2_ FC values were 6.85 in the microarray dataset (Supplementary Table 9). Consistent with our findings, a significant higher expression of lncRNA-ZFAS1 and DDX21 was observed in the vast majority of cancers, including CRC based on the TCGA dataset (Supplementary Figure 1B, 1C). Next, we demonstrated the expression levels of lncRNA-ZFAS1 and DDX21 in CRC cells and our included paired CRC patient’s tissues. As expected, the examination results were obtained that lncRNA-ZFAS1 and DDX21 showed significantly increased in CRC cells including LOVO, CACO2, HT29, SW48, HCT116, SW620, and SW480 compared with that in the normal control HIEC cells assayed by RT-PCR, qPCR method (Figure 1B, 1C). Similarly, RT-PCR and qPCR method confirmed the elevated expression of lncRNA-ZFAS1 and DDX21 between 20 pairs of CRC tissues and matched adjacent-tumor controls (Figure 1D, 1E), suggesting the possibly positive correlation of lncRNA-ZFAS1 with DDX21 in CRC cells and tissues. Thereafter, we enlarged the CRC patient samples to further clarify the expression association of lncRNA-ZFAS1 and DDX21, which included 157 pairs of CRC tissues and matched tumor-adjacent control tissues detected by ISH and IHC assay (Figure 1F). Notably, the expression levels of lncRNA-ZFAS1 and DDX21 were both up-regulated in the cases in comparison to matched controls (Figure 1G). Consequently, a positive linear correlation expression pattern was confirmed between lncRNA-ZFAS1 and DDX21 (*R*^2^ = 0.2795, *P* < 0.0001), shown in Figure 1H.

To further explore the clinical utility of the indicators as independent prognostic factors in this cohort, each index of low or high expression were determined by the cut-off point values assayed by ROC curve method in these included 157 pairs of CRC cases and control samples (Supplementary Figure 1D, 1E). Remarkably, the log-rank test prognostic analysis revealed that elevated lncRNA-ZFAS1 expression had significantly shortened DFS (*P* = 0.016; high expression: MST = 34 months, low expression: MST = 52 months) and OS (*P* = 0.025, high expression: MST = 35 months, low expression: MST = 60 months) (Figure 1I, 1J). Similarly, the higher expression of DDX21 protein was significantly associated with poor DFS (*P* = 0.010, high expression: MST = 36 months, low expression: MST =62 months) and OS (*P* = 0.029, high expression: MST = 30 months, low expression: MST = 50 months), shown in Figure 1I, 1J. Consistently, multivariate Cox regression analyses also further confirmed the independent prognostic values of lncRNA-ZFAS1 and DDX21 after adjusting for confounders including age and pathological pattern at diagnosis for OS and tumor stage for DFS (Supplementary Table 10). Additionally, the interactive effects of lncRNA-ZFAS1, DDX21 expression and the environmental factors, clinical variables were detected by unconditional logistic regression adjusted by gender, ages, tumor size and differentiation and so on. We found that the expression of ZFAS1 or DDX21 significantly correlated with the survival status of CRC patients including DFS and OS and without significant relationship observed with other clinical features, outlined in Supplementary Tables 1 and 2.

Collectively, our results indicated that lncRNA-ZFAS1 expression was up-regulated companied by its correlated potential target DDX21 up-regulation in human CRC cells and tissues. Furthermore, these two indicators could be considered as independent prognostic predictors for CRC prognosis evaluation.

### LncRNA-ZFAS1 mediated CRC molecular characteristics by regulating DDX21

**Figure 2:**
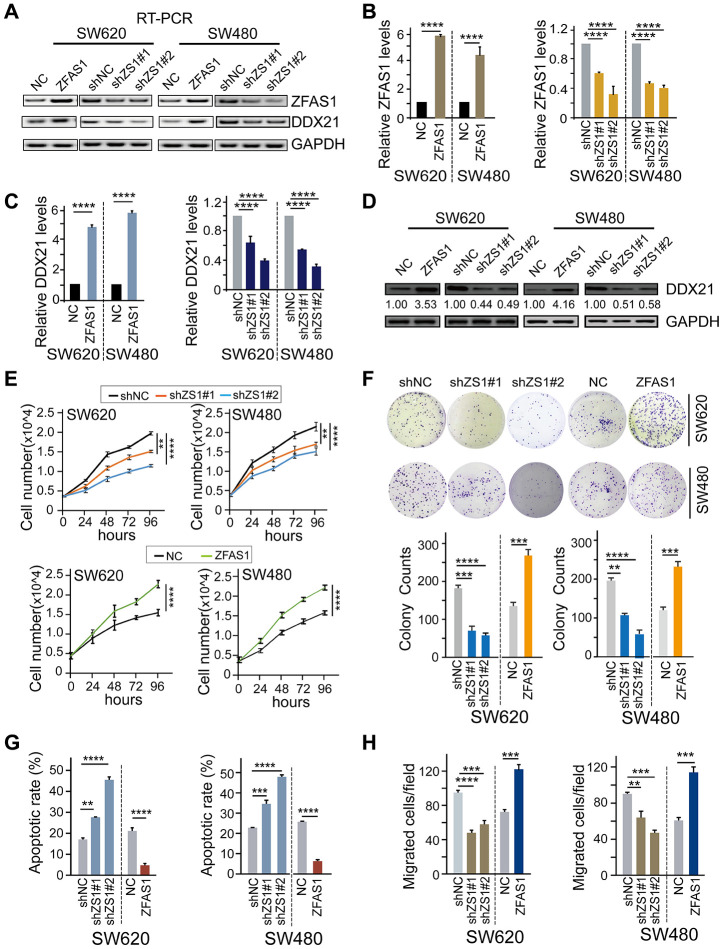
**The effect of lncRNA ZFAS1 on CRC cell biological characteristics by regulating DDX21.** (**A** and **B**) The expression of lncRNA ZFAS1 after overexpression or silencing lncRNA ZFAS1 in SW620 and SW480 cells by RT-PCR (**A**) and qPCR assay (**B**). (**C** and **D**) The mRNA and protein levels of DDX21 expression after interfering lncRNA ZFAS1 expression in both SW620 and SW480 cells detected by qPCR assay (**C**), and western blot (**D**). (**E**) Cell number monitoring assays showed the cell proliferative variation after ectopic or knockdown lncRNA ZFAS1 in SW620 and SW480 cells. (**F**) Cell colony formation assays were performed to identify the cell cloning capability upon lncRNA ZFAS1 silencing or overexpressing in SW620 and SW480 cells. *n* = 3 independent experiments. (**G**) The percentage (%) of cell apoptosis was detected upon lncRNA ZFAS1 overexpressing or silencing in SW620 and SW480 cells by Flow cytometry. *n* = 3 independent experiments. (**H**) The migration ability was determined after ectopic or knockdown lncRNA ZFAS1 in SW620 and SW480 cells. *n* = 3 independent experiments. Data were shown as mean ± s.d.. * *P* <0.05; **P <0.01; *** *P* <0.001; **** *P* <0.0001.

To further establish the regulation pattern of DDX21 and lncRNA-ZFAS1 involved in CRC biological characteristics, we firstly detected the expression levels of these two indicators after interfering lncRNA-ZFAS1 expression in both SW620 and SW480 cells. Our results demonstrated that ectopic *lncRNA-ZFAS1* expression significantly prompted the *DDX21* expression. Nevertheless, knockdown of *lncRNA-ZFAS1* resulted in inhibition of the *DDX21* expression both in SW620 and SW480 cells assayed by RT-PCR, qPCR, and western blot methods (Figure 2A–2D). Thereafter, cell number monitoring assays and colony formation assays (CFA) further revealed that lncRNA-ZFAS1 overexpression significantly enhanced the cell growth and colony formation capacity of SW620 and SW480 cells, whereas the lncRNA-ZFAS1 depletion suppressed the cell proliferative and colonic abilities of these two types of CRC cells (*P* < 0.0001, Figure 2E, 2F). Importantly, lncRNA-ZFAS1 depletion also resulted in an obvious elevated cellular apoptosis rates. In contrast, lncRNA-ZFAS1 overexpression substantially inhibited apoptosis rate both in SW620 and SW480 cells determined by flow cytometry, as shown in Figure 2G, and Supplementary Figure 2A. Moreover, Trans-well assay was conducted to detect the invasive abilities, and revealed that migrated cells of SW620 and SW480 cells were significantly suppressed while silencing lncRNA-ZFAS1 expression. Instead, ZFAS1 up-regulation dramatically elevated the invasive ability of SW620 and SW480 cells (Figure 2H, Supplementary Figure 2B). In addition, the cell proliferative abilities were inhibited and the cellular apoptosis rates were promoted after treated by DDX21 knockdown in SW620 and SW480 cells, shown in Supplementary Figure 2C, 2D.

**Figure 3:**
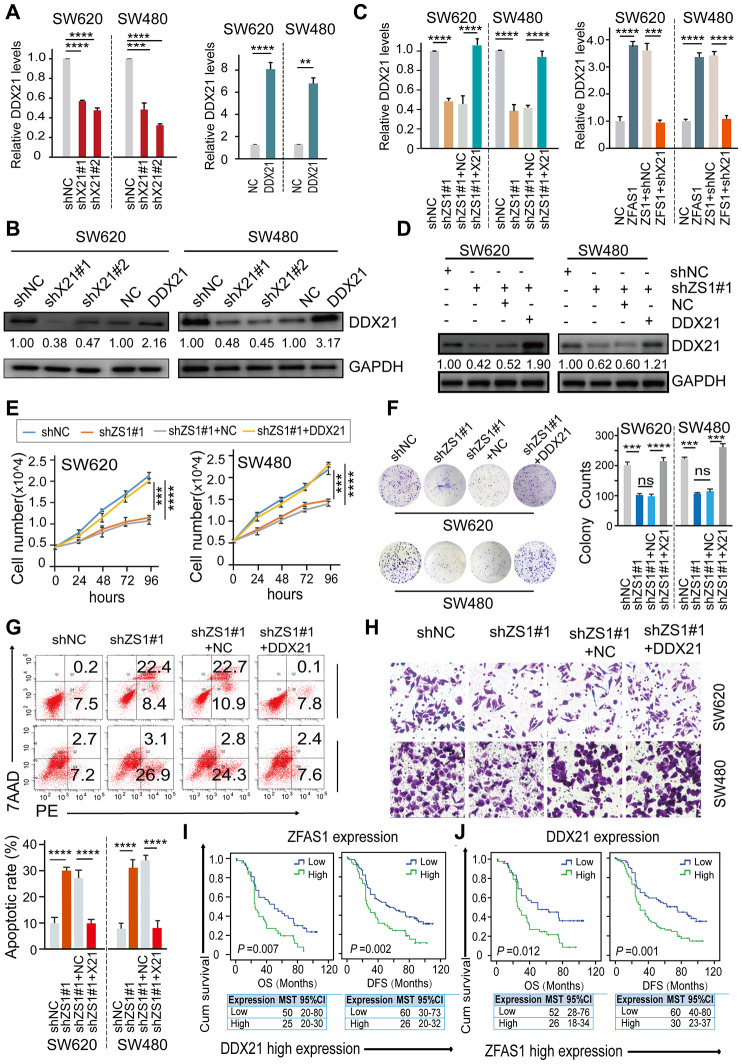
**DDX21 rescued the CRC cells proliferation inhibition caused by ZFAS1 knockdown.** (**A** and **B**) The expression of DDX21 after silencing or overexpression DDX21 in SW620 and SW480 cells by qPCR (**A**) and western blot (**B**). (**C** and **D**) Rescue experiments detecting the DDX21 mRNA and protein expression levels treated by co-transfection of lncRNA ZFAS1 silencing and DDX21 overexpression vectors or ZFAS1 overexpression with DDX21 silencing vectors in SW620 and SW480 cells assayed by qPCR assay (**C**) and western blot assay (**D**). (**E**–**H**) The cell proliferation, colony formation, invasion ability, and cellular apoptotic rates were recovered after co-transfected with *shRNA* ZFAS1 and pcDH-DDX21 vector in SW620 and SW480 cells assayed by cell number monitoring assay (**E**), cell colony formation assay (**F**), flow cytometry method (**G**), trans-well (**H**) respectively. (**I** and **J**) Stratified Kaplan-Meier plot illustrating the impact of ZFAS1 high/low expression on the DFS and OS upon those patients with DDX21 high expression (**I**), DDX21 high/low expression on the prognosis based on the patients with lncRNA ZFAS1 high expression (**J**). Data were shown as mean ± s.d.. *n* = 3 independent experiments. Two-tailed Student’s *t*-tests were used. *P <0.05; **P <0.01; ***P <0.001; ****P <0.0001.

To further establish the influence of lncRNA-ZFAS1 on regulating DDX21 and their possible biological function in CRC cells, we carried out rescue experiments by up-regulating DDX21 expression in lncRNA-ZFAS1 knockdown CRC cells. Primarily, we detected the expression of DDX21 at mRNA and protein levels after treated by silencing or overexpression of DDX21 in SW620 and SW480 cells assayed by RT-PCR (Supplementary Figure 3A), qPCR and western-blot method (Figure 3A, 3B). Furthermore, the mRNA and protein levels of DDX21 were testified to achieve recovery expression caused by ZFAS1 knockdown upon DDX21 or overexpression ZFAS1 on silencing DDX21 compared with the NC group determined by qPCR and WB assays both in SW620 and SW480 cells (Figure 3C, 3D). Similarly, *in vitro* rescue experiments revealed that DDX21 overexpression reversed the facilitated effect on CRC molecular characteristics including cell proliferation ability, cell colony formation capacity, cell migration ability, and apoptotic rates in both SW620 and SW480 cells assayed by cell number monitoring (Figure 3E), CFA assay (Figure 3F), flow cytometry analysis (Figure 3G) and Trans-well (Figure 3H and Supplementary Figure 3B), respectively. More importantly, stratified prognostic analysis in our included cohort (*n* = 157) revealed that in the subgroup of harboring DDX21 high expression patients, lncRNA-ZFAS1 high expression patients were significantly associated with a shorten OS (*P* = 0.007, high expression: MST = 25 months, low expression: MST = 50 months) and DFS (*P* = 0.002, high expression: MST = 26 months, low expression: MST = 53 months) (Figure 3I). Consistently, stratification of lncRNA-ZFAS1 high expression subgroup also confirmed the synergistic prognostic values on DDX21 high expression cohort showing a poor clinical outcomes including OS (*P* = 0.012, high expression: MST = 26 months, low expression: MST = 52 months) and DFS (*P* = 0.001, high expression: MST = 30 months, low expression: MST = 60 months) (Figure 3J).

Taken together, these findings supported that inhibition effect of CRC progression and development caused by lncRNA-ZFAS1 knockdown could be reversed by ectopic DDX21 expression. A further synergistic prognostic results strongly suggesting these indicators as precision biomarkers for CRC clinical prognosis prediction and evaluation.

### Identification of the direct interaction between lncRNA-ZFAS1 and DDX21 in CRC cells

**Figure 4:**
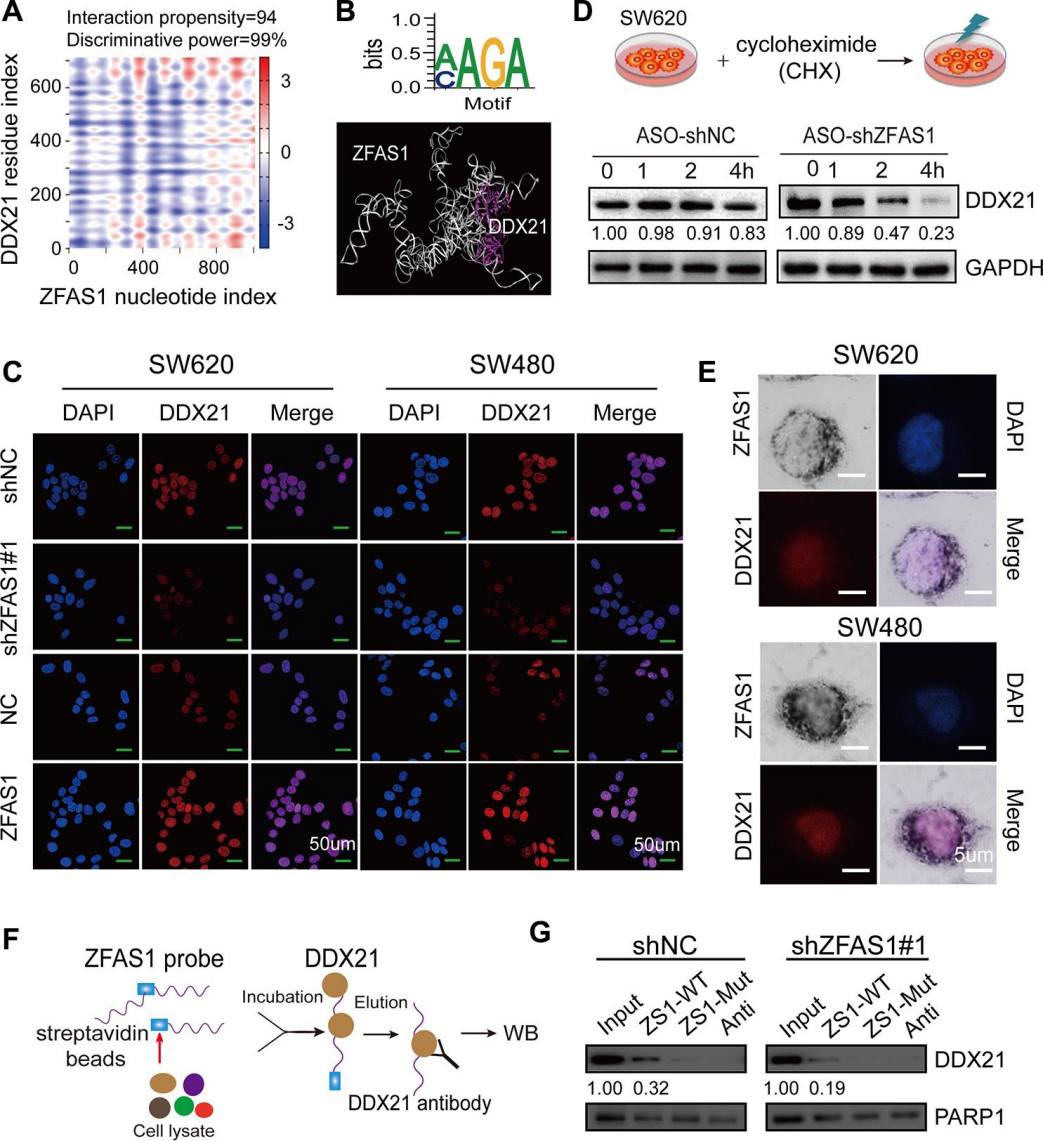
**Identification of the direct interaction between lncRNA ZFAS1 and DDX21 protein.** (**A**) Bioinformatics online database predicting the specific binding sequence and domain of lncRNA ZFAS1 secondary structure and DDX21 protein. (**B**) CLIP database illustrating the critical motif of lncRNA ZFAS1 (AAGC/CAGA) interacting with DDX21; MOE multi-functional docking platform showing the specific docking sites between ZFAS1 tertiary structure and DDX21 protein. (**C**) The DDX21 was determined the cellular localization and expression levels after knocking down or overexpressing lncRNA ZFAS1 in SW620 and SW480 cells by IF assays (**C**). Scale bar = 50μm. (**D**) The protein expression levels of DDX21 were determined by translation inhibition assay. (**E**) Co-localization of lncRNA ZFAS1 and DDX21 protein detected by the combination of ISH and IF assays in SW620 and SW480 cells. Scale bar = 5μm. (**G** and **F**) RNA pull-down followed by western blot showing the direct interaction of the ZFAS1-WT, ZFAS1-Mut, and antisense RNA probes with DDX21 protein after lncRNA ZFAS1 knockdown.

To further elucidate the underlying mechanism of DDX21 in the context of lncRNA-ZFAS1 mediated CRC development and its regulation manner, we employed catRAPID (http://service.tartaglialab.com) to evaluate the interaction propensity and power between the lncRNA-ZFAS1 nucleotide index and the DDX21 residue index (Figure 4A). As expected, the sequence (+383-+434) of lncRNA-ZFAS1 exhibited remarkably interaction propensity (value = 94) and discriminative power (99%) with the DDX21 based on secondary sequence (Figure 4A and Supplementary Table 13). The critical binding motif were also predicted by *POSTAR2* (http://lulab.life.tsinghua.edu.cn/postar/), illustrated in Figure 4B. Furthermore MOE software identified the direct binding motif (AAGA or CAGA) of ZFAS1 with DDX21 protein (RF classifier = 0.85, SVM classifier = 0.74) (Figure 4B). Thereafter, co-expression of lncRNA-ZFAS1 with DDX21 results identified that lncRNA-ZFAS1 knockdown results in the inhibition of DDX21 expression. In contrast, overexpression of lncRNA-ZFAS1 dramatically enhanced DDX21 expression both in SW620 and SW480 cells detected by IF techniques (Figure 4C). Importantly, we employed the protein translation inhibition assay treated by the cycloheximide (CHX). As expected, the protein expression levels of DDX21 were gradually decreased dependent on this time course (0, 1, 2, 4 hours) (Figure 4D) in SW620 cells. Of note, cellular co-localization of these two indicators substantially revealed that lncRNA-ZFAS1 and DDX21 distributed consistently in the cellular nucleus of SW620 and SW480 CRC cells assayed by combination of ISH and IF assays (Figure 4E). Finally, we deigned and synthesized the biotin-labeled lncRNA-ZFAS1 probe based on the sequence (+383-+434) of lncRNA-ZFAS1 containing the key binding sites with DDX21 (ZFAS1-WT) or containing a corresponding mutant sequence (ZFAS1-Mut) for the further RNA pull down assay (Figure 4F and Supplementary Table 14). Subsequently, the result further demonstrated that the ZFAS1-WT probe, but not ZFAS1-Mut, significantly pulled down endogenous nuclear DDX21 protein (Figure 4G), and this enrichment was significantly decreased when treated by ZFAS1 knockdown vector, indicating a direct interaction with the DDX21 protein.

Thus, these data supported that ZFAS1 regulated DDX21 expression by direct binding manner through specific recognizing motif, and this recruitment significantly impacted CRC tumorigenesis and development.

### POLR1B is a critical target of DDX21 regulated by ZFAS1 in CRC cells and tissues

**Figure 5:**
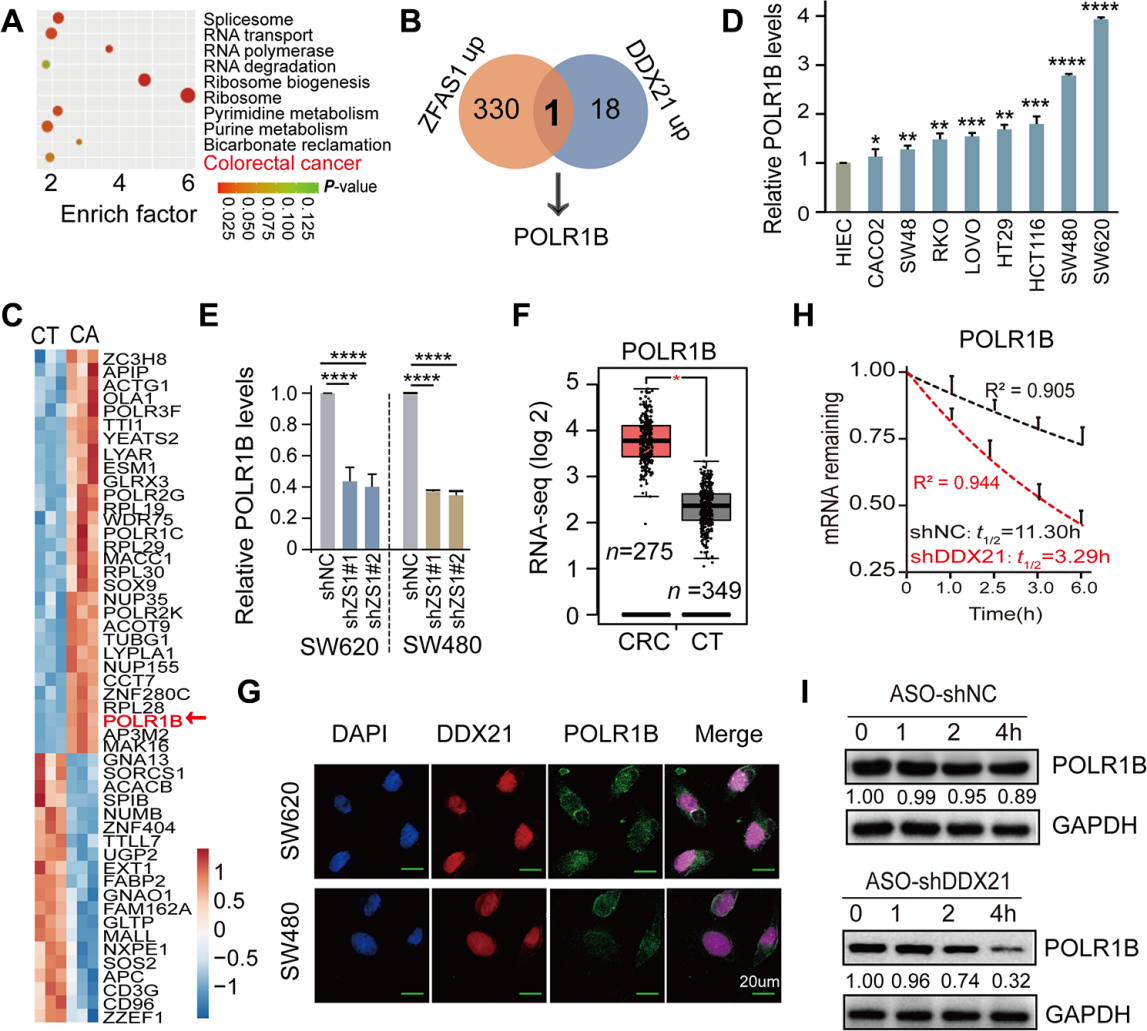
**POLR1B is a critical target of lncRNA ZFAS1 interacting with DDX21.** (**A**) KEGG and GO analysis enriched the co-expression target genes and the top 10 cellular function components affected by lncRNA ZFAS1 and DDX21. (**B**) The intersection of co-expression downstream genes between lncRNA-ZFAS1 up-regulation and DDX21 up-regulation. (**C**) The hierarchical clustering heat map showing the DDX21 related target genes in CRC patient tissues and their matched paired adjacent-tumor samples (*n* = 3). Red in heat map denotes up-regulation. Blue denotes down-regulation. (**D**) The expression of POLR1B in CRC cells including SW480, SW620, HCT116, SW48, CACO2, LOVO, HT29, and RKO cells and in normal intestinal epithelial HIEC cell assayed by the qPCR method. GAPDH was selected as an internal control. (**E**) The mRNA expression of POLR1B and POLR1A after lncRNA ZFAS1 knockdown in SW620 and SW480 cells by qPCR assay. (**F**) RNA-seq data showing the log 2 gene expression of POLR1B in CRC patients tissues (*n* = 275) and the normal controls (*n* = 375) based on the TCGA dataset (http://gepia.cancer-pku.cn/). (**G**) Co-localization of POLR1B protein and DDX21 protein detected by IF assays in SW620 and SW480 cells. Scale bar = 20μm. (**H**) RNA stability assay detected the POLR1B mRNA decay after knockdown DDX21 expression. (**I**) The protein expression levels of POLR1B were measured by translation inhibition assays treated with CHX. Data were shown as mean ± s.d.. Two-tailed Student’s *t*-tests were used. *P <0.05; **P <0.01; ***P <0.001; ****P <0.0001.

To further excavate the downstream regulation axis of DDX21 involved in lncRNA-ZFAS1 functions in CRC cells and tissues, we employed the enrichment of co-expression target genes and the top 10 cellular function components including RNA transport, RNA polymerase, and colorectal cancer analyzed by KEGG and GO analysis (Figure 5A). It was noteworthy that POLR1B was exactly appeared in the intersection of co-expression downstream genes between lncRNA-ZFAS1 up-regulation and DDX21 up-regulation (Figure 5B). Consistently, STRING website (https://string-db.org/) was applied for searching with potential downstream target genes of DDX21 protein. Interestingly, the POLR1B, the critical components of Pol I RNA polymerase complex, occurred in the DDX21 protein interaction networks, as shown in Supplementary Figure 4A. Furthermore, the heat-map cluster also identified a dramatic up-regulation of POLR1B based on our *Affymetrix GeneChip* microarray (*n* = 3) (Figure 5C, Supplementary Table 11). Similar results further established that POLR1B expression was significantly increased in these included CRC cells such as SW480, SW620, HCT116, SW48, CACO2, LOVO, HT29, and RKO cells compared with HIEC cells assayed by qPCR and RT-PCR method (Figure 5D, Supplementary Figure 4B). Further qPCR assay provided evidence that POLR1B was significant regulated by interfering ZFAS1 expression both in SW620 and SW480 cells (Figure 5E). A consistent expression pattern of POLR1B was also confirmed in majority of cancers including colorectal cancer upon the TCGA dataset (http://gepia.cancer-pku.cn/) (Figure 5F, Supplementary Figure 4C). Co-localization and co-expression assayed subsequently detected the similarly cellular distribution and localization of POLR1B and DDX21, which mainly located in the cellular nucleus of SW620 and SW480 cells (Figure 5G). Moreover, we conducted the RNA stability assays with the treatment of actinomycin D (*ActD*). As expected, the half-life of POLR1B mRNA decay was reduced after knockdown DDX21 expression in SW620 cells (Figure 5H). In agreement with these findings, the protein expression levels of POLR1B were measured after treatment with the CHX, and we observed that the expression levels gradually decreased throughout this time course (0, 1, 2, 4 hours) in SW620 cells (Figure 5I).

Collectively, these results implied that POLR1B might be a critical biomarker and candidate direct target of LncRNA-ZFAS1 and DDX21 in CRC cells, however, its underlying molecular mechanism need to be investigated.

### LncRNA-ZFAS1-DDX21-PLOR1B signaling axis in CRC cells and tissues

**Figure 6:**
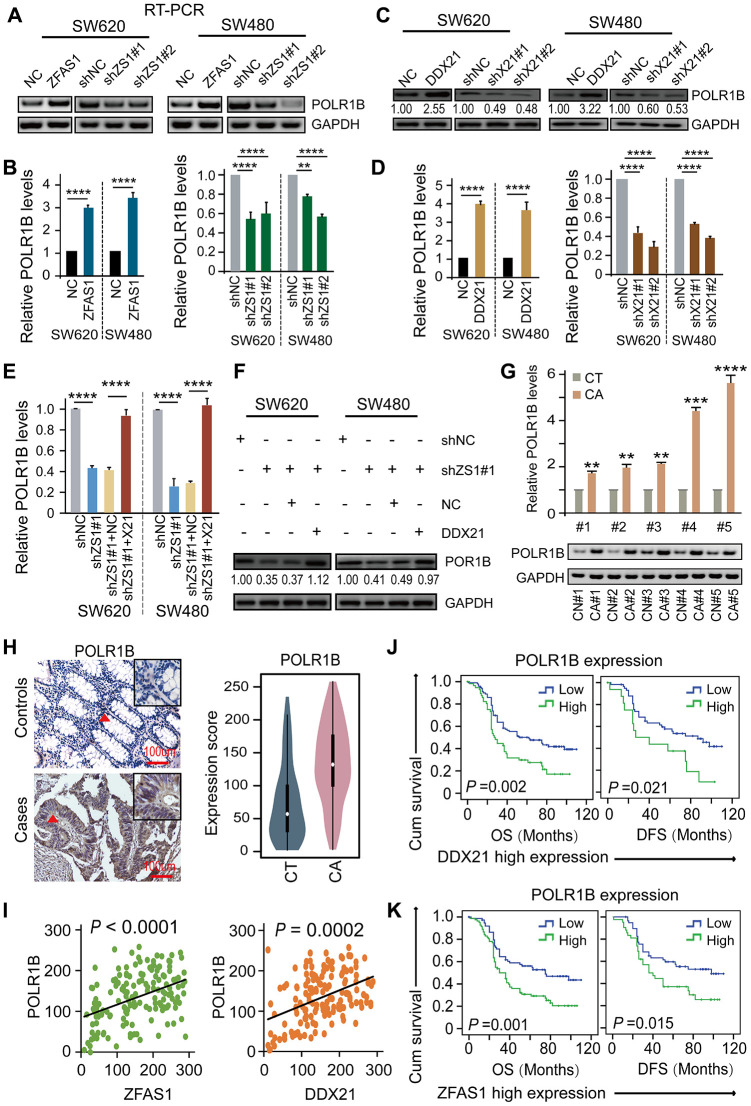
**DDX21-PLOR1B signaling axis regulated by lncRNA ZFAS1 in CRC cells and tissues.** (**A** and **B**) The mRNA expression of POLR1B after overexpression or silencing lncRNA ZFAS1 in SW620 and SW480 cells by RT-PCR (**A**) and qPCR assay (**B**). (**C** and **D**) The expression levels of POLR1B after interfering DDX21 expression in both SW620 and SW480 cells detected by western blot (**C**) and qPCR assay (**D**). (**E** and **F**) Rescue experiments determining the POLR1B mRNA and protein expression levels treated by silencing of lncRNA ZFAS1 and overexpression of DDX21 in SW620 and SW480 cells assayed by qPCR assay (**E**) and western blot assay (**F**). (**G**) Representative analysis results of POLR1B expression in paired CRC tissues and controls assayed by RT-PCR and qPCR method. 5 representative data was shown. (**H**) Representative IHC imagines and Violin charts displaying the POLR1B protein expression based on the CRC patient tissues and matched tumor-adjacent controls (*n* = 157). The bar represents 100μm. (**I**) Linear regression analysis illustrating a positive correlation between the expression of POLR1B and LncRNA ZFAS1 or DDX21. (**J** and **K**) Stratified Kaplan-Meier plot illustrating the impact of POLR1B high/low expression on the DFS and OS upon those patients with DDX21 high expression (**J**) or with ZFAS1 high expression (**K**). Data were shown as mean ± s.d. Two-tailed Student’s *t*-tests were used. *P <0.05; **P <0.01; ***P <0.001; ****P <0.0001.

To elucidate the underlying critical role of POLR1B regulated by DDX21 and lncRNA-ZFAS1, we therefore examined the impact of lncRNA-ZFAS1 and/or DDX21 on POLR1B expression in CRC cells and paired matched CRC tissue samples. Specifically, overexpression of lncRNA-ZFAS1 dramatically prompted the mRNA and protein expression, however, lncRNA-ZFAS1 knockdown resulted in an inhibition of the POLR1B expression in both SW620 and SW480 cells detected by qPCR and western blot assay (Figure 6A, 6B). Consistent with these results, ectopic DDX21expression dramatically enhanced POLR1B expression, whereas DDX21 depletion remarkably deceased the POLR1B expression at mRNA and protein levels in SW620 and SW480 cells (Figure 6C, 6D). Importantly, based on exhibited combined pcDH-DDX21 and ZFAS1 silencing vector, POLR1B expression was almost rescued to the normal levels at both RNA and protein expression, illustrated in Figure 6E, 6F. Consistent with the results obtained from the CRC cells, significant high expression of POLR1B was observed in 20 pairs of CRC tissues and matched tumor-adjacent controls using qPCR and RT-PCR assay (Figure 6G). Thereafter, we detected the expression of POLR1B, and assessed possibly correlation with LncRNA-ZFAS1 or DDX21 in this included relative large samples containing 157 paired of CRC tissues and matched tumor-adjacent control tissues by TMA and IHC assays (Figure 6H). As expected, an obviously increased POLR1B expression was determined in CRC tissues based on the cut-off values assessed by ROC curve method (Figure 6H, Supplementary Figure 5A). Of all clinicopathological features considered for this cohort, we found that the high expression of POLR1B was significantly associated with the CRC tumor size (*P* = 0.002), indicating high expression of POLR1B was related to malignant progression in CRC patients, shown in Supplementary Table 12. More importantly, a positive linear correlation regression manner was confirmed between POLR1B and lncRNA-ZFAS1 expression and DDX21 expression (Figure 6I). Notably, Kaplan-Meier analysis indicated that high expression of POLR1B was markedly associated with worse DFS (*P*=0.001) and OS (*P*=0.002) (Supplementary Figure 5B). The independent prognostic value of POLR1B expression was further established in the multivariate Cox proportional hazard model after adjusting for confounders (Supplementary Table 10). More importantly, stratified prognostic analysis in our included cohort (*n* = 157) revealed that POLR1B high expression showed a dramatically shorten OS (*P* = 0.002) and DFS (*P* = 0.021) in the subgroup of DDX21 high expression patients (Figure 6J, Supplementary Figure 5C). Similarly, stratification of ZFAS1 high expression subgroup also confirmed the synergistic prognostic values of POLR1B high expression cohort showing a poor clinical outcomes including OS (*P* = 0.001) and DFS (*P* = 0.015) (Figure 6K, Supplementary Figure 5C). Therefore, the findings strongly supported that a critical novel ZFAS1-DDX21-POLR1B signaling axis exerted their molecular function by involvement of CRC initiation and development.

### DDX21 promoting cell proliferation regulated by LncRNA-ZFAS1 *in vivo*

**Figure 7:**
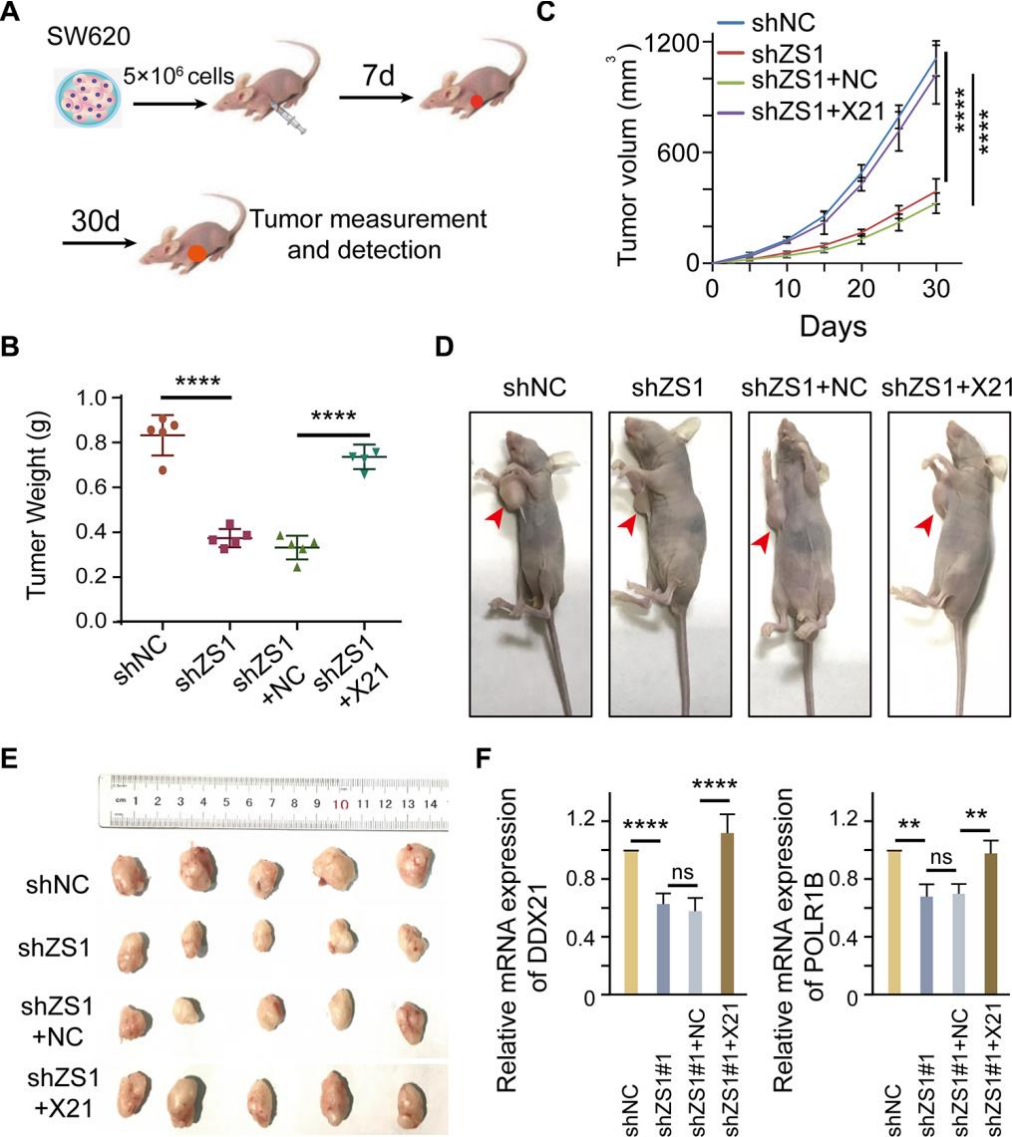
**DDX21 promoting cell proliferation regulated by LncRNA-ZFAS1 *in vivo.*** (**A**) Schematic diagram of xenografts in BALB/c nude mice by inoculating SW620 cells that were stably co-transfected with *shZFAS1*, *shZFAS1*+ *pcDH*-DDX21, *shNC* (empty-vector), and *shZFAS1*+*NC* at their right armpits. (**B**) Mean xenografts tumor weight for each group was determined on the 30^th^ day. Data are shown as mean ± s.d., *n* = 5 for each group. (**C**) Mean tumor volumes on different days for each group xenografts in nude mice. (**D** and **E**) Representative xenograft mice and tumors excised on the 30^th^ day are shown. (**F**) qPCR assays were performed to determine the mRNA expression of DDX21 and POLR1B above each group. The groups were as follows: *shNC* (empty vector); *shZS1* (*shZFAS1*#1); *shZS1*+NC (co-transfected with *shRNA* and *pcDH* empty vector); *shZS1*+X21 (co-transfected with *shZFAS1* and *pcDH*-DDX21).*, *P* <0.05; **, *P* <0.01; ***, *P* <0.001; **** *P* <0.0001.

To evaluate the molecular function of ZFAS1 interacting with DDX21 *in vivo*, we established xenografts in BALB/c nude mice by inoculating SW620 cells that were stably transfected with shZFAS1, shZFAS1+ pcDH-DDX21, empty-vector (shNC), and shZFAS1+NC. A total of 5×10^6^ cells were subcutaneously implanted into the right armpits of the four-week male mice (Figure 7A). The mice were sacrificed, and the xenograft tumors were removed at the sixth week after implantation. *Ex vivo* assessment of xenograft tumor weight and volume showed a significant change between the treated mice and the control mice after 4 weeks of growth (Figure 7B–7E). Specifically, ZFAS1 depletion results in a dramatically suppression in tumor weight at the days of 30 compared with the NC group. However, the reduced tumor weight almost recovered to the levels of NC group after transfected with pcDH-DDX21 vector in the knocking-down ZFAS1 xenograft tumor mice (Figure 7B). Similarly, the volume of xenograft tumor was monitored once every 5 days and the results showed than ZFAS1 silencing inhibited the growth speed or development of the SW620 cells in xenografts, nevertheless, no significant difference of the tumor volume were determined between *shZFAS1*+pcDH-DDX21 group and *shNC* group (Figure 7C), and the representative nude mice and all of the xenograft tumors exhibited in Figure 7D and 7E. Thus, knocking-down ZFAS1 and then overexpressing DDX21 tumors displayed the promotion of cell growth *in vivo*. To further confirm this, qPCR assay indicated that DDX21 was significantly increased in the group co-transfected with *shZFAS1* and DDX21 vector. Importantly, the target gene POLR1B mRNA expression showed a significantly elevated by the presence of *shZFAS1* and DDX21 (Figure 7F), indicating the promotion is a result of ZFAS1 interacting with DDX21in xenograft mice tumors. Therefore, the results demonstrated that ZFAS1 knockdown inhibits CRC cell proliferation for targeting DDX21/PLOR1B signaling axis *in vivo*.

## DISCUSSION

In humans, CRC remains a major public health issue as the third cause of cancer morbidity and mortality worldwide [32]. Deficiencies of the effective screening biomarkers often caused the recurrence of the CRC patients although improved treatment of surgical resection, chemotherapy, and/or radiotherapy [33, 34]. More and more research supported that lncRNAs have been highlighted as novel biomarkers for cancer diagnosis and prognosis [16, 35, 36], even as tumor potential therapeutic targets due to their involvement in multiple biological processes including cell proliferation and differentiation, RNA binding or decay [18, 37]. However, the underlying molecular mechanism of the candidate indicators and their related regulation network remains elusive, which needs to be further investigated, especially in CRC.

Here, we uncovered the lncRNA ZFAS1-DDX21-POLR1B regulatory axis involved in the initiation and development of CRC *in vitro* and *in vivo*. Specifically, our present study was the first to demonstrate the positive regulation of nucleolar RNA helicase DDX21, POLR1B (a key subunit of Pol I complex) with ZFAS1 and showed synergistically impact on prognosis in a relative large CRC cohort (*n* = 157). Importantly, lncRNA-ZFAS1 exerted its function as an oncogene by direct binding DDX21 through harboring the specific motif (AAGA/CAGA) and affected its downstream target gene POLR1B stability and translation, which subsequently promoted CRC cell proliferation, migration, invasion, and suppressed cell apoptosis.

It is worthy to note that DDX21, an ATP-dependent RNA helicase, involves ribosome RNA processing, transcription, and modification [7, 15, 38, 39]. However, a clearly defined mechanism about DDX21 is still in its infancy. In our present study, we provided profound insights into our understanding of the effects of DDX21 and highlighted its potential importance and versatility in promoting tumorigenesis and the development of CRC. Of note, our study for the first time to determine the association of DDX21 and ZFAS1 and evaluate the prognostic values of these indicators at the overall and stratification levels. In CRC cells and tissues, DDX21 and its endogenous regulatory RNA-ZFAS1 were both up-regulated. *In vitro* rescue experiments showing that DDX21 overexpression reversed the inhibition effect on CRC cell proliferation, migration caused by ZFAS1 knockdown in both SW620 and SW480 cells. Further studies in larger cohort assist in confirming that DDX21 and ZFAS1 were synergistic prognostic biomarkers for CRC clinical prognosis evaluation. Stratified prognostic analysis in our included cohort (*n* = 157) revealed that ZFAS1 high expression was significantly associated with a poor clinical outcomes, especially in these patients with DDX21 high expression, Consistently, lncRNA-ZFAS1 high expression subgroup also confirmed the synergistic prognostic values on DDX21 high expression cohort showing a poor prognosis including OS and DFS. Contrary to our results, a study of breast cancer displayed that DDX21 low expression was correlated with higher metastasis and poor clinical prognosis, and knockdown of DDX21 promoted breast cancer cells epithelial-mesenchymal transition (EMT), invasion and migration *in vitro* and *in vivo* [40]. However, a bioinformatics analysis of large clinical data determined CRC potential biomarkers including DDX21 and its association with clinicopathological features, which showed identical with our results [41]. Analogously, Cao et al reported that DDX21 was up-regulated in the gastric cancer tissues and cells, which induced the cell growth by up-regulating the Cyclin D1 and CDK2 expression [42]. Based on these discoveries, we derived that the expression levels of DDX21 showed tumor heterogeneity in various tumors and the molecular mechanism of DDX21 need to be precisely clarified in different cancers.

Recently, critical lncRNAs were found to act as protein activators by direct binding to the specific domain of recruited proteins so as to regulate their activity or change the cellular localization, thereby playing a pivotal role in the progression of malignant tumors [19, 43–45]. In this study, our findings identified that ZFAS1 functions as a critical oncogene in CRC and that its expression and function were required for both development and maintenance in CRC cells and tissues. Mechanistically, we for the first time reported that DDX21 protein was recruited by direct binding with ZFAS1 through recognizing the specific motif AAGA or CAGA. These results were supported by bioinformatics prediction of 2D or 3D structure, and the functional experiments including co-localization of ISH and IF, RNA pull-down assays. Although this regulation pattern of RNA-protein interaction had been widely investigated [46–48], however, no study was reported between ZFAS1 and DDX21 in regard to cancers. In osteosarcoma, ZFAS1 was observed directly interacting with ZEB2 protein and then regulated the stability of ZEB2, thereby leading to increased cell growth and metastasis [49]. Another research reported that ZFAS1 directly bound to SERCA2a protein, and impaired systolic function and cardiomyocyte apoptosis [50]. Therefore, it is plausible that designing lncRNAs inhibitors or activators, protein inhibitors or activators could provide novel therapeutic targets for various diseases, particularly malignant tumors.

Pol I complex, a multi-subunit enzyme contains 14 polypeptide subunits in eukaryotes, plays essential roles in the regulation of ribosomes RNA (rRNA) transcription and synthesis [13, 51, 52]. POLR1B is one of the largest subunits of Pol I complex and associated with clinical outcomes of multiple types of human cancer including non-small cell lung cancer, lymphoma [53, 54], but not been identified in CRC yet. In our present study, POLR1B was demonstrated dramatically up-regulated in CRC cells and tissues. Importantly, high expression of POLR1B was significantly associated with worse DFS and OS when in comparison with low POLR1B expression in CRC patients, which was previously unknown in CRC patients. Furthermore, POLR1B was identified as a novel downstream direct target of DDX21 regulated by ZFAS1. To elucidate the underlying critical functions of POLR1B involved in DDX21 and LncRNA-ZFAS1 expression, we examined the effect of ZFAS1 and/or DDX21 on POLR1B expression in CRC cells and paired CRC tissues. As expected, ectopic DDX21 or ZFAS1 expression dramatically enhanced POLR1B expression, whereas DDX21 or ZFAS1 depletion suppressed the POLR1B expression at mRNA and protein levels. Furthermore, POLR1B expression was rescued to the normal levels when treated with pcDH-DDX21 and ZFAS1 knockdown vector. Notably, a positive linear regression was confirmed between POLR1B and ZFAS1 and DDX21 expression. The independent prognostic value of POLR1B expression was established in the log-rank test and the multivariate Cox proportional hazard model. Synergistic prognostic values of POLR1B high expression cohort showing a worse clinical outcome in the stratified subgroup of DDX21 or ZFAS1 high expression cohort. Thus, our study strongly supported that a critical novel ZFAS1-DDX21-POLR1B signaling axis exerted the molecular function by the involvement of CRC initiation and development.

In summary, our work uncovers a previous unrecognized ZFAS1-DDX21-POLR1B signaling axis in colorectal cancer. By direct bound to DDX21, ZFAS1 recruits DDX21 protein through the specific motif (AAGA or CAGA) and subsequently affecting their downstream target gene POLR1B. This, in turn, mediates CRC cell proliferation promotion and apoptosis inhibition *in vitro* and *in vivo*. Our research provides insights into an unreported molecular mechanism of the lncRNA ZFAS1 in the regulation of CRC initiation and pathogenesis, which provide novel potential biomarkers and therapeutic targets for CRC treatment and prognostic evaluation.

## MATERIALS AND METHODS

### Clinical patient specimens

In the present study, the human specimens were included based on 157 pairs of clinical CRC tissue specimens and matched adjacent-tumor controls tissues from the First Hospital of China Medical University, and Cancer Hospital of China Medical University between September 2014 and September 2015. All of the cases were clearly diagnosed by histopathology and were excluded from the history of other malignant tumors, and received no chemotherapy or radiation before surgery. All of the patients signed the written informed consent form before enrolled this study. Furthermore, the study was approved by the Medical Ethics Committee of China Medical University. These specimens collected from patients were immediately snap frozen in liquid nitrogen and stored at -80°C before using. The clinicopathological characteristic of these included patients are outlined in Supplementary Tables 1 and 2.

### LncRNAs-mRNAs microarray assay

The GeneChip^®^ Human Transcriptome Array 2.0 (HAT2.0, Affymetrix, USA) was explored containing more than 6.0 million distinct probes covering coding and non-coding transcripts of human genomes at Shanghai OE Biotech Technology Co, Ltd (Shanghai, China). We obtained the accession number GSE137511 by deposited the raw data to the Gene Expression Omnibus (GEO) (https://www.ncbi.nlm.nih.gov/geo/query/acc.cgi?acc=GSE137511). Databases including Ensembl, UCSC, NONCODE, RefSeq, lncRNAdb, Vertebrate Genome Annotation (Vega), Mammalian Gene Collection (MGC), and Human Body Map lincRNAs were selected to annotate the determined transcripts. The data were analyzed with Robust Multichip Analysis (RMA) algorithm using Affymetrix default analysis settings and global scaling as normalization method.

### Gene expression analysis

Genesrping software (version 13.1; Agilent Technologies) was employed to perform the raw data analysis. Deferentially expressed genes were then identified through fold change as well as *P* value calculated with *t*-test. The threshold of up- and down-regulated genes was set at fold change ≥ 1.5 and *P* ≤ 0.05. Afterwards, gene ontology (GO) enrichment analysis and Kyoto Encyclopedia of Genes and Genomes (KEGG) analysis were applied to determine the roles of these deferentially expressed mRNAs played in these GO terms or pathways. Finally, Hierarchical Clustering was performed to display the distinguishable genes’ expression pattern among the included 8 samples.

### Cell lines and cell culture

The normal human intestinal epithelial cell line HIEC, and CRC cell lines includingSW480, SW620, HCT116, SW48, RKO, CACO2, LOVO, and HT29 were purchased from Peking Union Medical College Cell Resource Center (PUMCCRC, Beijing, China). HIEC, HCT116, LOVO, CACO2, RKO, and HEK293T cells were cultured in Dulbecco’s Modified Eagle’s Medium (DMEM, Invitrogen, USA). SW480, SW620, and SW48 cells were grown in L15 medium (Hyclone, USA), and HT29 cells were maintained in 5A medium (5A, Invitrogen, USA) with 10% fetal bovine serum (PAA, Germany) at 37°C with 5% CO2 atmosphere. In this study, all cells were determined for absence of mycoplasma contamination.

### Cell transfection

Plasmid extraction kit was obtained from Sangon Biotech (Shanghai, China). The short hairpin RNA (*shRNA*) for silencing ZFAS1 (*shZFAS1#1*, *shZFAS1#2*) and the negative control (*shNC*) were purchased from Genepharma (Shanghai, China). The pc*DNA-ZFAS1*, pc*DNA-DDX21* and blank vector (*NC*) were synthesized by Genewiz (Suzhou, China). All of the *shRNA* nucleotide sequences were listed in Supplementary Tables 3, 4. Cells were cultured on 6-well plates to 60%-70% density and then transfected by using Lipofectamine 2000 (Invitrogen, USA) according to the manufacturer’s instructions. The cells were used for further experiments after 48 hours of transfection.

### Reverse transcript-PCR and Quantitative real-time PCR assays

In brief, total of RNAs were extracted from CRC cells and CRC patient tissues by *TRIzol* reagent (Invitrogen, USA) following the manufacturer’s instructions. The cDNA was synthesized using a qPCR RT Kit (TOYOBO, Japan). Thereafter, the RT-PCR analyses were performed by using RT kit Quant Studio^®^ 3 (Thermo, USA). qPCR were conducted using SYBR Green Real-time PCR Mix (Toyobo, Japan) and were determined in triplicate. The relative mRNA expression of genes was quantified to GAPDH mRNA. The relative expression level of the gene was calculated using the 2^*−ΔΔCt*^ method. The primers used for RT-PCR and qPCR amplification were listed in Supplementary Tables 5, 6.

### Western blot analysis

Cultured cells were washed twice in cold phosphate-buffered saline (PBS) and lysed by 1×SDS buffer. Lysates were sonicated and centrifuged (13000 rpm, 4°C) for 10 min. Proteins concentration were detected and equal amounts of protein were separated by 10%-12% SDS-PAGE and transferred to polyvinylidene fluoride membranes (Millipore, Bedford, MA). Membranes were immunoblotted with anti-rabbit DDX21 (NB100-1718, 1:2000, Novusbio, USA), POLR1B (PA5-48383, 1:1000, Thermo Fisher, USA) and anti-mouse GAPDH (1:2000, Zsbio, China), after sealed with 2% BSA, and the membranes were incubated with hybrid secondary antibody, the data was statistically collected by Fluor Chem V2.0 (Alpha Innotech Corp, USA).

### Immunofluorescence (IF) assay

Briefly, cells were seeded into 24-well plates containing glass cover slips, fixed with methanol, blocked, and stained with the corresponding antibodies. The antibodies used for immunofluorescence including DDX21 (NB100-1718, 1:200, Novus, USA), Alexa Fluor anti-rabbit IgG (#4412, 1:500, Cell Signaling Technology), Alexa Fluor anti-mouse IgG (#8890, 1:500, Cell Signaling Technology), Alexa Fluor anti-Rat IgG (#4417, 1:500, Cell Signaling Technology). The secondary Antibodies were incubated at room temperature for 1 h. Cell nuclei were counterstained with DAPI (Beyotime, Shanghai, China). All image acquisition was processed using a Nikon C2 plus confocal microscope under a 40×objective (Nikon, Japan).

### Co-localization of lncRNA and protein expression

Cells were cultured on cover slides and fixed normally following above the steps of IF assay. Then RNA *in situ* hybridization was also performed following the kit instructions above except counterstaining with 0.1% Hematoxylin. Next, cell was continued to stain with indicated DDX21 antibody, Alexa Fluor anti-rabbit IgG and DAPI. Finally, Nikon C2 plus confocal microscope was used to obtain image under a 40 × objective (Nikon, Japan).

### Cell number monitoring assay

Firstly, wiped the cell counting plate and coverslip cleanly, covered the coverslip on the counting plate. Then, absorbed a little dilution (10 ul) of the cell suspension and injected it on the edge of the coverslip to fill the suspension between the coverslip and the counting plate. Then cells were observed under microscope, after that, calculated the total number of cells in the four large cells of the counting plate. The crimped cells only counted the left and upper cells, and finally calculated the total number of cells according to the corresponding formula.

### Apoptosis analysis

The transfected cells (1×10^6^) were harvested and washed twice with cold 1×PBS and stained by using an AnnexinV-PE/7-AAD kit (BD Biosciences, USA) according to the manufacturer’s protocol. Briefly, cells were resuspended with 100μL of Annexin V binding buffer and incubated with 5μL of annexin V-PE and 5μL of 7-AAD for 15min in a darkroom at room temperature. Finally, the apoptosis rate of cells was detected by flow cytometry analysis and analyzed using a BD FACSCanto II system (BD Biosciences, USA).

### Trans-well assay

The cell invasion and migration assay was conducted by using 24-well insert Trans-well chambers (Corning Costar, USA). The transfected cells (1×10^4^/well) were firstly suspended in serum-free medium and then added to the upper chambers or Matrigel-coated Trans-well insert. By contrast, the bottom chamber medium contained 10% FBS. After 48 hours, the upper chamber was removed and cells on the filter membrane were wiped off with cotton swabs. Chambers were washed with PBS, then fixed in 4% Paraformaldehyde for 20 minutes, washed with PBS, air-dried. After that, chambers were stained with 0.1% crystal violet solution for 10 min. After washing with distilled water, invaded and migrated cells were captured in 5 random fields under a microscope and we finally counted the cell number.

### Tissue microarray (TMA) and immunohistochemistry (IHC)

TMA and IHC method was performed as previous described. Briefly, Paraffin donor blocks containing representative colorectal cancer samples and adjacent normal tissues were selected by reviewing the hematoxylin and eosin-stained slides. Tissue cores with a diameter of 1.5 mm were extracted from each donor block, and precisely arrayed into a new paraffin recipient block with a maximum of 55 cores using the Organization Microarrayer (Pathology Devices, USA). Sections (4 μm) were deparaffinized with xylene, rehydrated in a graded alcohol series, and washed in distilled water. Then, sections were incubated in primary antibody of DDX21 (NB100-1718, 1:100, Novusbio, USA), POLR1B (PA5-48383, 1:100, Thermo Fisher, USA) overnight at 4°C, followed by incubation with biotinylated secondary antibodies for 30 min at 37°C. The slides were incubated with horseradish peroxidase coupled streptavidin for additional 30 min (LSAB kit; Dako, Glostrup, Denmark), and stained with DAB (3, 3-diaminobenzidine). Sections were counterstained with hematoxylin, dehydrated, and mounted. Protein expression levels were observed and counted under a microscope (Eclipse 8i, Nikon, Japan) for subsequent analysis.

### Tissue *in situ* hybridization method

*In situ* hybridization was performed strictly following the kit instructions (Boster, Wuhan, China). Before prehybridized in prehybridization solution at 42°C for 2h, slides were deparaffinized and deproteinated, then incubated with DIG-labeled probe solution (dilute 4 times with 1×PBS) at 37°C overnight (specific sequences of probes for ZFAS1 were showed in Supplementary Table 7). After stringent washing, the slides were exposed to a streptavidin-peroxidase reaction system and and stained with DAB (Zsbio, Beijing, China) for 2 min. Then 0.1% Hematoxylin (Solarbio, beijing, China) was used to counterstain the slides for 5 min. LncRNA expression levels were observed and counted under a microscope (Nikon, Tokyo, Japan).

### RNA pull-down assay

HEK293T cells with or without ZFAS1 knockdown seeded in 10 cm dish at 70-80% confluency were cross-linked by UV and harvested by trypsinization. Nuclear extraction was isolated by 500 μl 1× hypotonic buffer and 10% NP-40. 40μM of ZFAS1-wild biotin labeled probe, ZFAS1-mutant biotin labeled probe, ZFAS1-antisense probe (negative control) were conjugated to Streptavidin agarose resin beads (Thermo Fisher Scientific) by incubation for 4 hours at 4 °C, respectively, followed by 3wash and incubation with pre-cleared nuclear extraction in RIP buffer [150 mM KCl, 25 mM Tris (pH 7.4), 5 mM EDTA, 0.5 mM DTT, 0.5% NP40, 1x protease inhibitor] at 4 °C overnight. After washing with RIP buffer for three times, followed by protein isolation with 40μl 1×SDS protein lysis at 95°C for 10 min and 13000g centrifuged for 10 min. Input and co-immunoprecipitated proteins were analyzed by SDS-PAGE separation, and the expression level of DDX21 were measured with PARP1as internal control.

### RNA stability assay

SW620 cells were transfected with *shNC*, *shDDX21#1*, followed with a treatment by actinomycin D (CAS#:A4262, Sigma) at a final concentration of 5 μg/mL for 0.5, 1, 2, 3, and 6 hours. Total RNA was extracted and analyzed by qRT-PCR. Then, the calculation of RNA turnover rate and half-life (*t*_1/2_) of POLR1B were determined according to the previous publications [55]. Since actinomycin D treatment results in transcription stalling, the change of RNA concentration at a given time (*dC/dt*) is proportional to the constant of RNA decay (*K*_decay_) and RNA concentration (*C*) as shown in the following equation:

$$dC/dt=-k_{decay}C$$

Thus the RNA degradation rate *k_decay_* was estimated by:

$$ln\;(C/C_{0})=-k_{decay}t$$

When 50% of RNA is decayed (i.e., *C/C_0_*=1/2), the equation below can be used to calculate the RNA half-life (*t*_1/2_):

$$ln(1/2)=-k_{decay}t_{1/2}$$

From where:

$$t_{1/2}=ln2/k_{decay}$$

### Translation inhibition assay

Briefly, the SW620 cells were cultured for one dish at each time point and then transfected with ASO-shNC, ASO-shZFAS1, ASO-shDDX21. After 48 hours, the cells were treated with translation inhibitor, cycloheximide (CHX, CAS#:C7698, Sigma) with a concentration of 200 μg/ml in the fresh cell medium. Thereafter, the cells were incubated with CHX based on the different time points (0, 1, 2, 4 hours). The zero hour represents the start time of treatment with CHX. The total protein was isolated according to the time courses. Finally, the expression levels of DDX21, POLR1B were measured with GAPDH as the internal control assayed by western blot method.

### Xenograft mice experiment

All mice were purchased from Shanghai Laboratory Animal Center (Shanghai, China). Before the experiments, the mice were acclimatized to the new environment for one week. 4-week-old BALB/c-nu mice were weighed. 5×10^6^ SW620 stably cells were subcutaneously injected into the right armpit region. All protocols used followed the Regulations of Experimental Animal Administration issued by the Ministry of Science and Technology of the People's Republic of China. When the tumors were visible (the tumor size reached to 50 mm^3^), the mice were randomly divided into four groups (5 mice for each group). The mice weight and tumor size were measured every 5 days with vernier caliper. Simultaneously, the survival of mice was tracked and recorded. 37 days after injection, the mice in each group were sacrificed and the subcutaneous tumors were isolated and measured. Also, the tumor tissues were fixed in 10% formalin for further research.

### Statistical analysis

All of the statistical analysis was employed using SPSS 19.0 software package (SPSS Inc. Chicago, USA), and GraphPad Prism 5 software (GraphPad, USA). The data are presented as mean ± standard deviation (*s.d.*) or median (quartile). Student’s *t* test or Wilcoxon T test was performed to analyze the significance differences of the paired and unpaired continuous variables. Pearson *χ*^2^or Fisher’s exact test was conducted to analyze the expression or distribution differences of the variables. Kaplan-Meier method and multivariate Cox proportional hazard regression analysis was used to estimate the potential prognosis associated indicators. *P-*values were two sides, and *P* < 0.05 was considered statistically significant in all tests.

### Ethics approval

This study was approved by the Ethics Committee of China Medical University. All the animal experiments performed in this study were approved by the Institutional Animal Care and Use Committee of China Medical University.

## Supplementary Material

Supplementary Figures

Supplementary Tables
