# Supplementary material for: Long non-coding RNA ZFAS1 promotes colorectal cancer tumorigenesis and development through DDX21-POLR1B regulatory axis
Source: Aging (Albany NY). 2020 Nov 16;12(22):22656–87. doi: 10.18632/aging.103875 (PMC7746388; doi:10.18632/aging.103875)
Supplement: Supplementary Figures [file aging-12-103875-s001..pdf]

SUPPLEMENTARY TABLES

Supplementary Table 1. Correlation between ZFAS1 expression and clinicopathological features in the included colorectal cancer patients (*n* = 157).

Characteristics	*n* =157	ZFAS1 expression		*P*-value	Adjusted OR(95%CI)
		Low (%) n=100	High (%) *n*=57		
**Age**					
<=64	77	46(59.7)	31(40.3)	0.325	
>64	80	54(67.5)	26(32.5)	0.242	1.404(0.725-2.720)
**Gender**					
Male	86	54(62.8)	32(37.2)	0.868	
Female	71	46(64.8)	25(35.2)	0.941	0.975(0.498-1.908)
**Family History**					
No	143	92(64.3)	51(35.7)	0.576	
Yes	14	8(57.1)	6(42.9)	0.560	0.713(0.229-2.219)
**Differentiated Degree**					
Low	85	55(64.7)	30(35.3)	0.590	
Medium	60	39(65.0)	21(35.0)	0.925	0.967(0.476-1.964)
High	12	6(50.0)	6(50.0)	0.126	0.355(0.094-1.337)
**Primary Organ**					
Colon	75	45(60.0)	30(40.0)	0.408	
Rectum	82	55(67.1)	27(32.9)	0.228	1.512(0.772-2.962)
**Infiltration**					
Negative	128	77(60.2)	51(39.8)	0.057	
Positive	29	23(79.3)	6(20.7)	0.059	2.545(0.966-6.701)
**Size**					
<=6	86	60(69.8)	26(30.2)	0.096	
>6	71	40(56.3)	31(43.7)	0.080	0.551(0.283-1.075)
**DFS**					
0 = progression	48	37(77.1)	11(22.9)	**0.030**	
1 = death	109	63(57.8)	46(42.2)	**0.012**	0.365(0.166-0.805)
**OS**					
0 = alive	58	44(75.9)	14(24.1)	**0.017**	
1 = death	99	56(56.6)	43(43.4)	**0.008**	0.369(0.176-0.774)

Abbreviations: 95%CI, 95% confidence interval; DFS, Disease-free survival; OS, Overall survival.
P values, Adjusted HR (95%CI) were assessed using *Pearson* χ^2 test and logistic regression analysis adjusted for age, differentiation.

Supplementary Table 2. Correlation between DDX21 expression and clinicopathological features in the included colorectal cancer patients (*n* = 157).

Characteristics	*n* =157	DDX21 expression		*P*-value	Adjusted OR(95%CI)
		Low (%) n=100	High (%) *n*=57		
**Age**					
<=64	77	33(42.9)	44(57.1)	0.631	
>64	80	38(47.5)	42(52.5)	0.564	1.205(0.639-2.274)
**Gender**					
Male	86	37(43.0)	49(57.0)	0.629	
Female	71	34(47.9)	37(52.1)	0.729	1.121(0.588-2.137)
**Family History**					
No	143	68(47.6)	75(52.4)	0.090	
Yes	14	3(21.4)	11(78.6)	0.066	0.287(0.076-1.088)
**Differentiated Degree**					
Low	85	39(45.9)	46(54.1)	0.899	
Medium	60	26(43.3)	34(56.7)	0.702	0.876(0.446-1.723)
High	12	6(50.0)	6(50.0)	0.977	0.982(0.282-3.419)
**Infiltration**					
Negative	128	54(42.2)	74(57.8)	0.148	
Positive	29	17(58.6)	12(41.4)	0.113	1.941(0.856-4.401)
**Size**					
<=6	86	43(50.0)	43(50.0)	0.201	
>6	71	28(39.4)	43(60.6)	0.185	0.648(0.341-1.232)
**DFS**					
0 = progression	48	31(64.6)	17(35.4)	0.002	
1 = death	109	40(36.7)	69(63.3)	0.001	0.287(0.139-0.595)
**OS**					
0 = alive	58	34(58.6)	24(41.4)	0.013	
1 = death	99	37(37.4)	62(62.6)	0.006	0.385(0.195-0.762)

Abbreviations: 95%CI, 95% confidence interval; DFS, Disease-free survival; OS, Overall survival.
P values, Adjusted HR (95%CI) were assessed using *Pearson* χ^2 test and logistic regression analysis adjusted for age, differentiation.

Supplementary Table 3. ***Short hairpin RNAs (shRNAs)*** **sequence against ZFAS1.**

shRNA	Sequence (5'-3')
ZFAS1-Homo-455	GCCATTCGTTCTTTCGCGTCT
ZFAS1-Homo-525	GCTATTGTCCTGCCCGTTAGA
ZFAS1-Homo-769(shRNA-1)	GATTCAGTCTGCCTTGTAACA
ZFAS1-Homo-884(shRNA-2)	CAAGGTTACTGTATACATAGC

Supplementary Table 4. ***Short hairpin RNAs (shRNAs)*** **against DDX21.**

shRNA	Sequence (5'-3')
DDX21-Homo-457(shRNA-1)	GGAGCCTTCTGAGGAAGAAAT
DDX21-Homo-933	GCAAAGACTTCAGTGACAYCA
DDX21-Homo-1245	GGGTATTTAATGTTGCCAAGA
DDX21-Homo-1983(shRNA-2)	GCTCCTTGATCAACTCAAATG

Supplementary Table 5. Reverse transcription polymerase chain reaction (RT-qPCR) assays.

mRNA and LncRNA	
**Relative reagents**	**1×system (10μl)**
5×RT Buffer	2 μl
Enzyme Mix	0.5 μl
Primer Mix	0.5 μl
RNA+DEPC H_2O	7 μl (400ng)
**Program: 37℃/15min→ 98℃/5min→ Maintain at 4℃**	
**qPCR**	
**Relative reagents**	**1×system(10μl)**
SYBR	5 μl
ROX	0.2 μl
Forward Primer(10μM)	0.4 μl
Reverse Primer(10μM)	0.4 μl
cDNA	4 μl
**Program: 95℃/60sec→[95℃/15sec→ 60℃/15sec→72℃/45sec]×40cycles→ Maintain at 4℃**	

Supplementary Table 6. Primers used in qRT-PCR assays.

Primer	Sequence (5'-3')
ZFAS1-Forward	GCTATTGTCCTGCCCGTTAG
ZFAS1-Reverse	TCGTCAGGAGATCGAAGGTT
DDX21-Forward	GGCAGCGTGTTCTGATTCTT
DDX21-Reverse	GCCTGCCAGAAGCTGTAGAGT
POLR1A-Forward	ACAAGCCAGTTCACCGTCTTCATG
POLR1A-Reverse	CTGTCCTCTCCACTCTGTCCTTCC
POLR1B-Forward	GTGGAGTCCTTCAACTACGCTGTG
POLR1B-Reverse	CTGCCTCCTCATGGTGCTCAATG
POLR1C-Forward	ACTATGGCTGTGGAGAAGGTCCTG
POLR1C-Reverse	GCCTTCTTCATCTCCTTGGTTCCG
POLR1D-Forward	TGCCTGGCTGTCCTCAGATCATC
POLR1D-Reverse	CGGTGGCGGAGAGGTCTATTCTAG
POLR1E-Forward	TTGTGGCTGCTGCTGTCTTAACTC
POLR1E-Reverse	CACCTCGCACTCGGCAACAC
GAPDH-Forward	CTCTGCTCCTCCTGTTCGAC
GAPDH-Reverse	ACCAAATCCGTTGACTCCGA

Supplementary Table 7. Probes used in situ hybridization (ISH) assay.

Digoxin-labeled probe	Sequence (5'-3')
ZFAS1-(1)	GGAACCCGTCGAGCGGTTTGGTGCGTGTGAAGCGACAT
ZFAS1-(2)	GGTTATATAAGGGAGGTTCAGGAAGCCATTCGTTCTTG
ZFAS1-(3)	CTACAACCTTCGATCTCCTGACGAGTTTATTGTTGGCCAA

Supplementary Table 8. Data of lncRNAs cluster in Heatmap analysis.

Up-regulated		Down-regulated	
LncRNA	Fold Change	LncRNA	Fold Change
**ZFAS1**	**6.65**	RNF138P1	2.21
TERC	3.17	PSMD6-AS2	2.64
SNORD12C	5.71	PGM5P2	2.64
SNORD12B	7.42	NPY6R	4.92
SNORD123	2.39	NCF1C	2.02
SNORD12	9.02	NCF1B	2.27
SNORA71D	5.83	LOC285972	2.37
SNORA71C	12.02	LOC285878	4.13
SNORA71B	3.27	LINC00675	2.64
SNORA71A	17.16	LINC00641	3.06
SNORA65	4.82	LINC00294	2.24
SNORA31	3.90	IGKV2-29	8.77
SNORA22	4.87	GVINP1	3.54
SNORA21	3.98	FENDRR	4.01
SNORA15	2.10	FENDRR	4.01
SNORA10	2.24	FAM83H-AS1	2.16
SNHG8	3.45	DLEU1	2.34
SNHG17	2.11	BACE2-IT1	3.05
SCARNA9L	3.11	ANKRD36BP2	3.31
SCARNA23	3.12	ADAMTS9AS2	3.18
RPL13AP20	2.06	ABCC13	2.12
RNU5F-1	2.13		
RNU105A	2.25		
PROX1-AS1	2.29		
MGC32805	2.78		
LOC541471	2.56		
LINC00493	2.10		
LINC00273	2.46		
HSP90AB3P	2.18		
HSD17B7P2	2.14		
FEZF1-AS1	3.69		
CRNDE	3.88		
CMAHP	6.25		

Supplementary Table 9. Data of mRNAs cluster in Heat map analysis.

Up-regulated		Down-regulated	
**mRNA**	**Fold Change**	**mRNA**	**Fold Change**
ABCE1	2.81	ABCG2	14.70
AGO2	2.02	AGL	2.71
BTF3	1.86	AGPAT9	3.57
CMSS1	2.73	BTC	2.27
DCTD	1.54	BTNL8	5.01
DDIT4	2.21	CLDN8	4.93
DDX10	2.46	CLMN	2.97
**DDX21**	**6.85**	CNNM4	3.68
DDX31	2.08	EIF4E3	2.16
EIF3M	2.52	GUCA2A	4.05
EIF4A3	1.96	GUCA2B	10.52
GTF3C3	1.54	HSPB8	5.93
GTPBP4	2.59	ITLN1	3.58
HSP90AB1	2.55	KCNIP4	2.95
HSPA8	3.43	LARGE	1.50
HSPD1	3.88	MYL12B	1.71
ITGA2	8.29	MYLK	4.01
KCNH8	4.16	PRKAG2	1.77
KPNA7	1.71	PRKCB	3.08
LAMC2	6.64	ZNF625	1.72
LARP1	2.14		
MYC	7.64		
PRPF40A	1.65		
PRPF6	1.89		
RPL29	2.38		
RPL30	2.18		
RPL31	1.78		
XRN2	3.63		
YEATS2	1.64		
ZC3H8	2.08		

Supplementary Table 10. Multivariate Cox regression analysis these indicators for DFS and OS in this included colorectal cancer patients (*n* = 157).

Variables	DFS				OS			
	Total *n*	Events *n* (%)	Adjusted HR(95%CI)	*P*	Total *n*	Events *n* (%)	Adjusted HR(95%CI)	*P*
**All patients**								
**ZFAS1**								
Low	100	63(63.0)	1(reference)	-	100	56(56.0)	1(reference)	-
High	57	46(80.7)	1.936(1.301-2.880)	**0.001**	57	43(75.4)	1.976(1.306-2.990)	**0.001**
**DDX21**								
Low	71	40(56.3)	1(reference)	-	71	37(52.1)	1(reference)	-
High	86	69(80.2)	1.671(1.128-2.475)	**0.010**	86	62(72.1)	1.587(1.053-2.390)	**0.027**
**POLR1B**								
Low	75	43(57.3)	1(reference)	-	75	38(50.7)	1(reference)	-
High	82	66(80.5)	1.931(1.309-2.847)	**0.001**	82	61(74.4)	1.923(1.278-2.893)	**0.002**

Abbreviations: 95%CI, 95% confidence interval; DFS, Disease-free survival; OS, Overall survival.
P values, Adjusted HR (95%CI) were assessed using multivariate Cox regression analysis adjusted for age, differentiation.

Supplementary Table 11. Data of mRNAs cluster of target genes in Heat map analysis.

Up-regulated		Down-regulated	
**mRNA**	**Fold Change**	**mRNA**	**Fold Change**
ACOT9	1.76		
ACTG1	1.84		
AP3M2	2.09		
APIP	2.11		
CCT7	2.47		
ESM1	4.08	ACACB	3.42
GLRX3	2.05	APC	1.54
LYAR	2.48	CD3G	2.36
LYPLA1	2.51	CD96	3.68
MACC1	7.44	EXT1	1.66
MAK16	2.90	FABP2	5.26
NUP155	4.23	FAM162A	2.52
NUP35	1.78	GLTP	3.08
OLA1	2.25	GNA13	2.02
**POLR1B**	**2.73**	GNAO1	10.23
POLR1C	1.54	MALL	10.24
POLR2G	1.56	NUMB	1.79
POLR2K	2.21	NXPE1	5.56
POLR3F	1.67	SORCS1	1.68
RPL19	1.63	SOS2	1.97
RPL28	1.71	SPIB	1.90
RPL29	2.38	TTLL7	1.89
RPL30	2.18	UGP2	4.15
SOX9	7.29	ZNF404	1.58
TTI1	2.24	ZZEF1	3.50
TUBG1	1.83		
WDR75	2.80		
YEATS2	1.64		
ZC3H8	2.08		
ZNF280C	2.46		

Supplementary Table 12. Correlation between POLR1B expression and clinicopathological features in the included colorectal cancer patients (*n* = 157).

Characteristics	*n* =157	POLR1B expression		*P*-value	Adjusted OR(95%CI)
		Low (%) n=100	High (%) *n*=57		
**Age**					
<=64	77	33(42.9)	44(57.1)	0.264	
>64	80	42(52.5)	38(47.5)	0.228	1.473(0.784-2.765)
**Gender**					
Male	86	40(46.5)	46(53.5)	0.750	
Female	71	35(49.3)	36(50.7)	0.801	1.086(0.572-2.061)
**Family History**					
No	143	69(48.3)	74(51.7)	0.784	
Yes	14	6(42.9)	8(57.1)	0.696	0.801(0.263-2.441)
**Differentiated Degree**					
Low	85	42(49.4)	43(50.6)	0.576	
Medium	60	26(43.3)	34(56.7)	0.390	0.744(0.379-1.459)
High	12	7(58.3)	5(41.7)	0.725	1.253(0.356-4.405)
**Primary Organ**					
Colon	75	32(42.7)	43(57.3)	0.264	
Rectum	82	43(52.4)	39(47.6)	0.155	1.595(0.838-3.037)
**Infiltration**					
Negative	128	60(46.9)	68(53.1)	0.684	
Positive	29	24(33.8)	14(48.3)	0.642	1.212(0.539-2.726)
**Size**					
<=6	86	51(59.3)	35(40.7)	0.002	
>6	71	40(56.3)	31(66.2)	0.002	0.347(0.180-0.670)
**DFS**					
0 = progression	48	32(66.7)	16(33.3)	**0.002**	
1 = death	109	43(39.4)	66(60.6)	**0.001**	0.303(0.147-0.627)
**OS**					
0 = alive	58	37(63.8)	21(36.2)	**0.003**	
1 = death	99	38(38.4)	61(61.6)	**0.001**	0.325(0.163-0.647)

Abbreviations: 95%CI, 95% confidence interval; DFS, Disease-free survival; OS, Overall survival.
P values, Adjusted HR (95%CI) were assessed using *Pearson* χ^2 test and logistic regression analysis adjusted for age, differentiation.

Supplementary Table 13. Evaluation the interaction propensity of lncRNA ZFAS1with DDX21.

Protein region	RNA region	Interaction Propensity
126-177	383-434	94.14
501-552	383-434	91.71
126-177	33-84	77.15
126-177	108-159	75.33
501-552	33-84	74.84

Supplementary Table 14. RNA probes used for RNA pull-down assays.

Probe	Sequence(5'-3')
ZFAS1-WT	GATTTTGGAAGAGGGAGTCACCACTGGAC
ZFAS1-Mut	GATTTTGGTTCTGGGAGTCACCACTGGAC
ZFAS1-antisense	CTAAAACCTTCTCCCTCAGTGGTGACCTG
