# Supplementary material for: Long non-coding RNA ZFAS1 promotes colorectal cancer tumorigenesis and development through DDX21-POLR1B regulatory axis
Source: Aging (Albany NY). 2020 Nov 16;12(22):22656–87. doi: 10.18632/aging.103875 (PMC7746388; doi:10.18632/aging.103875)
Supplement: Supplementary Tables [file aging-12-103875-s002..pdf]

SUPPLEMENTARY FIGURES

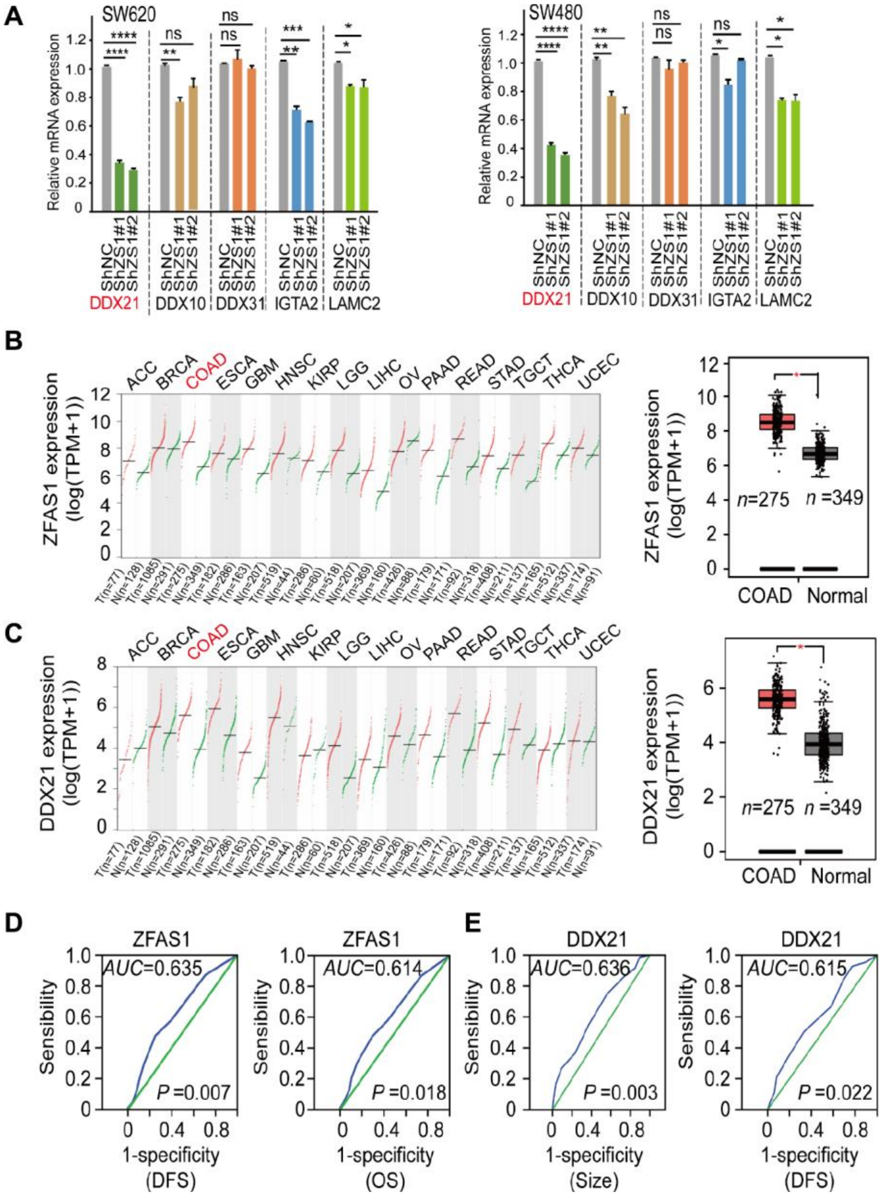

Supplementary Figure 1. The expression of lncRNA ZFAS1 and its correlation with DDX21 expression based on the TCGA datasets and our included paired CRC patient tissues. (**A**) The mRNA levels of potential target indicators were detected after silencing lncRNA ZFAS1 in SW620 and SW480 cells by qPCR assay. (**B** and **C**) RNA-seq analysis illustrating the log2 gene expression of lncRNA ZFAS1 and DDX21 in a variety of tumor tissues (left) and comparison the relative expression in CRC patients tissues (n = 275) and the normal controls (n = 375) (right) based on the TCGA data (http://gepia.cancer-pku.cn/). (**D** and **E**) The ROC curves illustrating the cutoff values of lncRNA ZFAS1 and DDX21 expression in paired CRC patient tissues vs. adjacent-tumor control tissues (n=157).

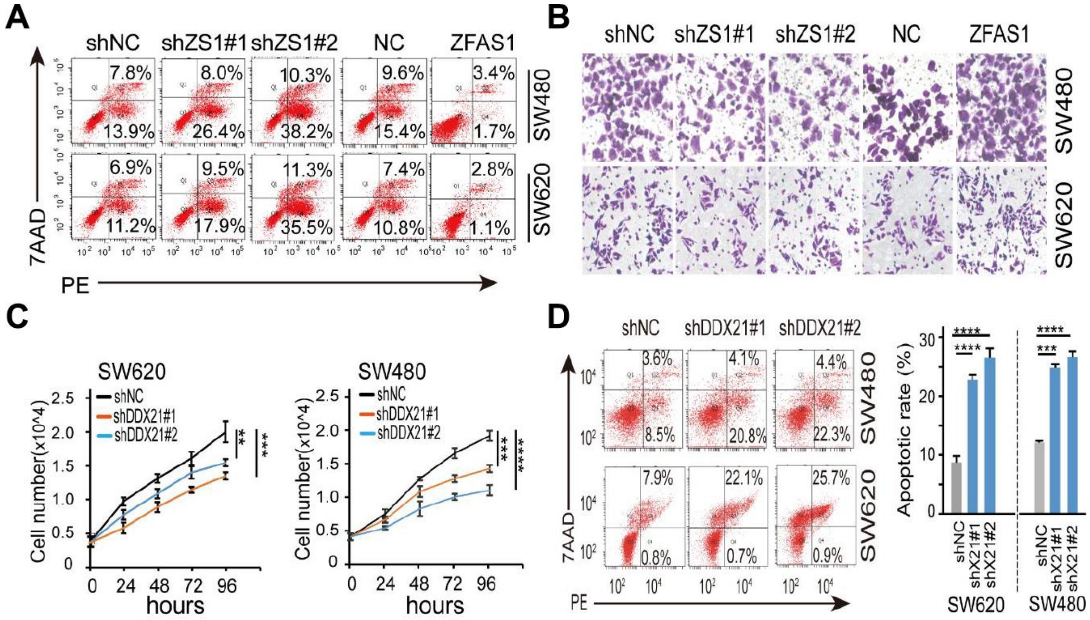

Supplementary Figure 2. The effect of lncRNA ZFAS1 and DDX21 on biological characteristics of CRC cells. (**A**) The cell apoptotic rates were determined after treated with lncRNA ZFAS1 overexpressing or silencing vector in both SW620 and SW480 cells by Flow cytometry. (**B**) The invasion ability was determined after ectopic or knockdown lncRNA ZFAS1 in both SW620 and SW480 cells. n = 3 independent experiments. (**C**) Cell number monitoring assays showed the cell proliferation after silencing DDX21. (**D**) Flow cytometry assays determined the cell apoptotic rates after DDX21 knockdown.

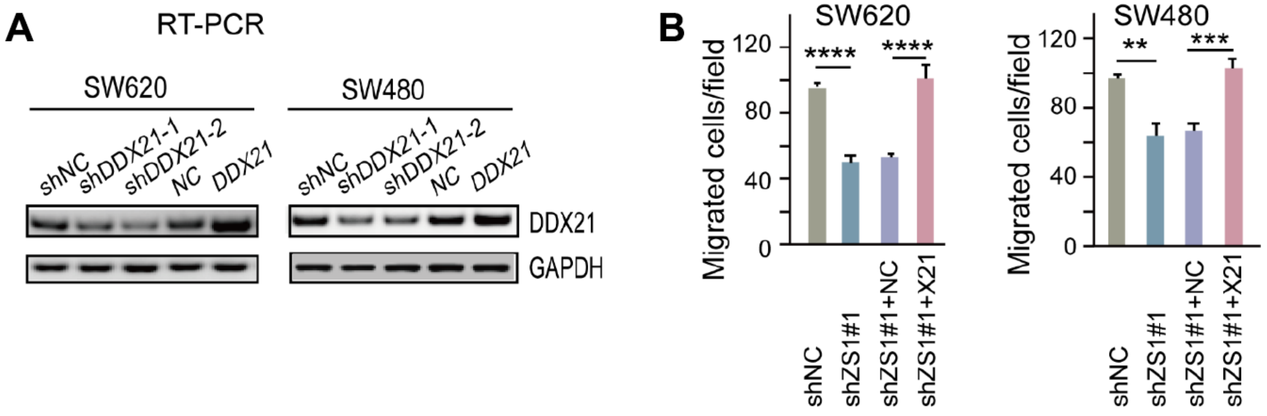

Supplementary Figure 3. DDX21 rescued the CRC cells proliferation inhibition caused by ZFAS1 knockdown. (**A**) The expression of DDX21 after silencing or overexpression DDX21 in SW620 and SW480 cells by RT-PCR assay. (**B**) The migrated cells per field were detected after co-transfected with shRNA ZFAS1 and pcDH-DDX21 vector in SW620 and SW480 cells using Trans-well assays.

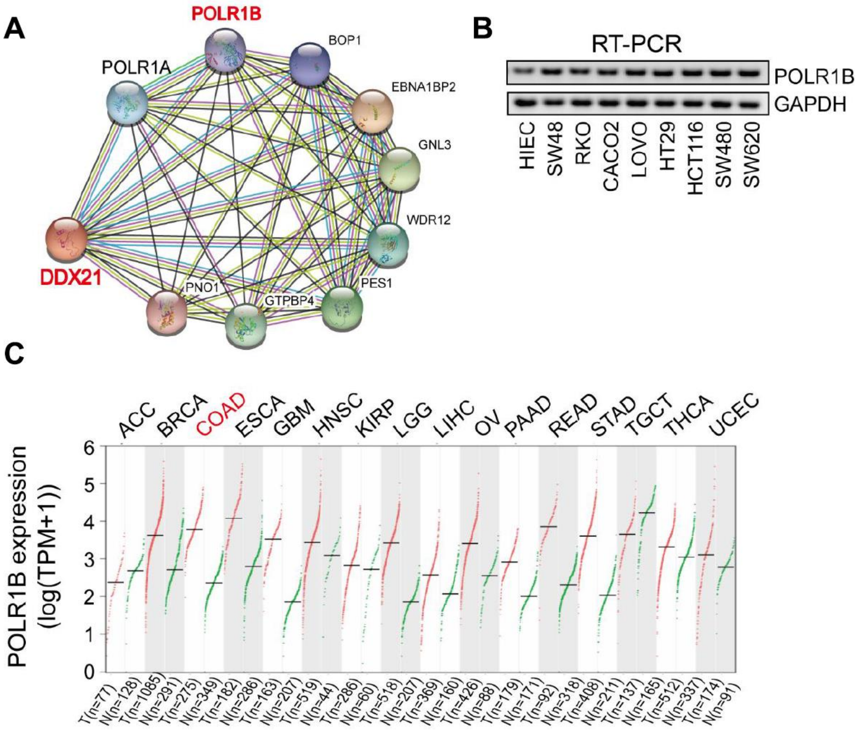

Supplementary Figure 4. The expression of POLR1B in the CRC cells and the TCGA datasets. (**A**) STRING (https://string-db.orgl) predicting the functional protein association networks of DDX21 including POLR1B. (**B**) RT-PCR assay detecting the POLR1B expression in CRC cells including SW480, SW620, HCT116, SW48, CACO2, LOVO, HT29, and RKO cells vs. normal intestinal epithelial HIEC cells. (**C**) RNA-seq analysis illustrating the log 2 gene expression of POLR1B in a variety of tumor tissues based on the TCGA data (http://gepia.cancer-pku.cn/).

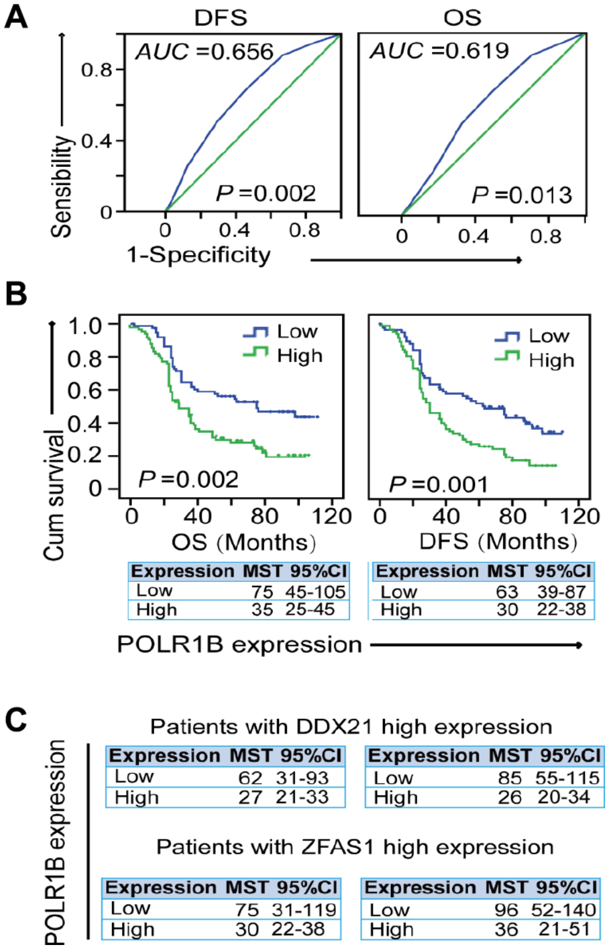

Supplementary Figure 5. Stratification analysis of the correlation of POLR1B with prognosis of CRC patients. (**A**) The ROC curves illustrating the cutoff values of POLR1B expression in paired CRC patient tissues vs. adjacent-tumor control tissues (n=157). (**B**) Kaplan-Meier plot representing the impact of POLR1B high/low expression on the DFS and OS in this cohort (n=157). (**C**) Stratified Log-rank test showing the MST and 95%CIs of POLR1B high/low expression on the DFS and OS based on the patients with DDX21 high expression (upper) or ZFAS1 high expression (lower).
